# Attenuating the nonresponse bias in hunting bag surveys: The multiphase sampling strategy

**DOI:** 10.1371/journal.pone.0213670

**Published:** 2019-03-15

**Authors:** Philippe Aubry, Matthieu Guillemain

**Affiliations:** 1 Cellule d’appui méthodologique, Direction de la Recherche et de l’Expertise, Office National de la Chasse et de la Faune Sauvage, Saint Benoist, BP 20, 78612 Le Perray-en-Yvelines, France; 2 Unité Avifaune Migratrice, Direction de la Recherche et de l’Expertise, Office National de la Chasse et de la Faune Sauvage, La Tour du Valat, Le Sambuc, 13200 Arles, France; University of Alberta, CANADA

## Abstract

Reliable hunting bag statistics are a prerequisite for sustainable harvest management based on quantitative modeling. Estimating the total hunting bag for a given game species is faced with a multiplicity of error sources. Of particular concern is the nonresponse error. We consider that the major cause of nonresponse bias is when the reluctance to respond is related to a null harvest, which leads to a potentially important overestimation. For tackling the nonresponse bias issue, we advocate the repeated subsampling of nonrespondents, with a final phase of personal interview by phone, intended to be without nonresponse. When a 100% response rate is actually reached at the last phase, both total and sampling variance can be estimated without bias, whatever the response rates at the previous phases. The actual case of imperfect response at the last phase is studied using Monte Carlo simulations. For imperfect response at the last phase, we show that the estimators we advocate are biased downwards but that these bias remain very moderate if the response rate at the last phase is high enough, depending on the circumstances. Furthermore, we illustrate that increasing the number of phases improves the nonresponse bias attenuation. In case of a hunting bag collecting scheme prone to a high nonresponse rate, for obtaining a very satisfying nonresponse bias attenuation we advocate relying on the multiphase sampling strategy with two- or three-phases, and a response rate in the last phase of at least 90%.

## Introduction

Management of harvested wildlife populations increasingly moves towards a science-based approach (but see [1–3]) where the sustainability of the populations and the hunting activity itself are ensured by adequate data collection (e.g. [4] for waterfowl in North America). Adaptive harvest management (see [5]) is increasingly used in this context, and relies on continuous monitoring of the populations and hunting bags as minimum required information [6]. Such management is often based on estimates of the population parameters, such as population size estimates based on counts of animals, or estimates of the total harvest. Until it is mandatory for hunters to report the total number of animals they hunt, even strong policy enforcement cannot lead to an absolute number of animals taken, and total harvest size is only estimated, often through questionnaires and deliberate will of the hunters to fill those.

In the context of hunting, the number of animals killed by a legal hunter (denoted *k*) is called a bag. Conditionally to given game species, spatial domain and time period (typically the hunting season), let *y*_*k*_ denote the bag for hunter *k* ∈ *U*, where *U* is the population of active hunters (an active hunter is one who participates in hunting, whether he/she is successful or not). We consider situations in which the parameter of interest is the total hunting bag $t=\sum_{k\in U}y_{k}=N\bar{y}$, with *N* the size of *U*. Knowing the total hunting bag at several geographical scales is needed for wildlife management, according to species biology (migratory or sedentary) and population status (threatened, of no concern, invasive, overabundant). Whatever the geographical scale considered, collecting hunting bag data may be achieved only imperfectly. Several reasons may be responsible for such a situation.

### 1.1 Response error

Hunting bag reporting may be affected by a response error, that is, a discrepancy between the correct number of animals killed for a given species, and that reported by the hunter. This error may be volunteer or not [7, 8]. The response error can be split into several components according to the origins of the error, for instance the prestige or pride to report higher than real bag [7, 9–11], or the inability to recall the exact value of the bag, leading to omission or digit preference [7, 9, 12–19]. Within a group of game animals, the direction of the response error may differ depending on the species, with overreporting for some of the common species, and underreporting for those that are less common [20]. Another possible component of the response error is the misclassification error, that is, attributing the bag to the wrong species, either because of misidentification [7, 21] or because of a name confusion due to regionalisms (see for instance the example mentioned in [22]). This type of error results in bias whose magnitude and direction depends on the species and the region of the country in which the hunters live (see [23], Table 10, and [24], p. 11). In addition, at moderate spatial scale, the hunter can make a location error by attributing the bag to the wrong spatial domain. Collecting hunting bag data is usually achieved by self-reporting, on paper or online questionnaires. In that case, a response error can simply arise through miss filling the correct line or column in the questionnaire (*reporting error*, or *mechanical error* in the sense of MacDonald & Dillman [10]). In practice, for a set of hunting bag data, these sources of response error are non-mutually exclusive from each other.

### 1.2 Sampling error

We assume that the total hunting bag is estimated by an estimator ${\hat{t}}_{s}$ calculated on the basis of a sample of hunters (denoted *s*), leading to a sampling error $\left({\hat{t}}_{s}-t\right)$. We consider that the sampling error is under the control of wildlife statisticians through the use of a probability sampling design *p*(⋅) (see for instance [25], Chapter 1). We note expectation and variance under the sampling design by using the subscript *p*, that is, E_*p*_(⋅) and V_*p*_(⋅). We refer in particular to *simple random sampling without replacement* (SRSWOR). Under SRSWOR, a design-unbiased estimator of *t* is ${\hat{t}}_{s}=N{\bar{y}}_{s}$, with ${\bar{y}}_{s}=n_{s}^{-1}\sum_{k\in s}y_{k}$, and *n_s_* is the size of *s*. The sampling error may be alternatively expressed in terms of means, omitting the factor *N*, that is $\left({\bar{y}}_{s}-\bar{y}\right)$. In what follows, we define the operator SRSWOR(*N*, *n*) for an SRSWOR sampling design involving a sample of size *n* drawn from a population of size *N*.

### 1.3 Coverage error

In the framework of probability sampling, for estimating the total hunting bag, a first concern is the coverage of the target population (that is, the active hunters). As expected, a poor coverage makes hunting bag surveys inefficient [26] and imperfect coverage results in surveys prone to biased estimations [27, 28]. It may actually be difficult to adequately cover the active hunter population for a given hunting season, and a given group of game species. Indeed, there may not be a full register of the hunters, especially in the absence of a hunting permit such as in the United Kingdom or Ireland for instance. When a register of hunters exists, generally a sub-register of potentially active hunters can be obtained, because the existence of a hunting permit is linked to a system of hunting licences, for all or part of the hunting season. Sometimes there exist legal provisions targeted at a group of game species upon which to rely to obtain a good coverage of the target population. For instance, in the U.S., the Migratory Bird Hunting and Conservation Stamp Act (in short, Duck Stamp Act) requires each waterfowl hunter 16 years of age or older to possess a valid Federal hunting stamp [4, 24, 29]. In this case, duck stamp purchasers form the population sampled (in practice, indirectly, i.e. through the duck stamp dealers). In the past, this frame have provided a rather good coverage of the population of active waterfowl hunters, with only 1% of stamp purchasers having no intention of hunting (see [24], p. 10, and [30]).

### 1.4 Nonresponse error

A major concern is the fact that only a subset of the sampled hunters respond to the survey. Such nonresponse leads to the partition *s* = *r* ∪ *m*, *r* ∩ *m* = ∅, where *r* of size *n*_*r*_ is the subset of respondents and *m* of size *n*_*m*_ is the subset of nonrespondents (*m* stands for *missing*). Consequently, under SRSWOR, the total estimator is now ${\hat{t}}_{r}=N{\bar{y}}_{r}$ with ${\bar{y}}_{r}=n_{r}^{-1}\sum_{k\in r}y_{k}$. In addition to sampling error, the nonresponse introduces another error:

$$\begin{array}{c} \underset{\text{total}\;\text{error}}{\underset{︸}{\left({\bar{y}}_{r}-\bar{y}\right)}}=\underset{\text{sampling}\;\text{error}}{\underset{︸}{\left({\bar{y}}_{s}-\bar{y}\right)}}+\underset{\text{nonresponse}\;\text{error}}{\underset{︸}{\left({\bar{y}}_{r}-{\bar{y}}_{s}\right)}} \end{array}$$ (1)

Whatever the nonresponse mechanism, the nonresponse bias can be written as:

$$\begin{array}{cc} \mathrm{NRBias}({\bar{y}}_{r}) & =E({\bar{y}}_{r}-\bar{y}) \end{array}$$ (2)

$$\begin{array}{cc} & =E({\bar{y}}_{r}-{\bar{y}}_{s}) \end{array}$$ (3)

$$\begin{array}{cc} & =E(n_{m}/n({\bar{y}}_{r}-{\bar{y}}_{m})) \end{array}$$ (4)

with ${\bar{y}}_{m}=n_{m}^{-1}\sum_{k\in m}y_{k}$. The expression (4) shows that if the nonresponse rate is not zero, then the bias depends on the difference between the means among respondents and nonrespondents. If the means are very close between respondents and nonrespondents, then the nonresponse bias may be neglected, even in case of a high nonresponse rate.

The estimator based on *r* may be unbiased only in case of ignorable missingness—for a thorough discussion about terminology, see [31] (pp. 103-106)—i.e. when the data are *missing completely at random* (MCAR) or *missing at random* (MAR) (see [32], p. 133). In the case of hunting bag surveys, given the difficulty to implement a proper sampling frame, generally gathering relevant auxiliary variables related to the hunting bag (or to the response propensity) is almost hopeless. Consequently, ignorable missingness is generally limited to the cases where the values taken by variable *y* are not related to the fact of being respondent or nonrespondent, i.e. MCAR mechanism (see [33], p. 7 and p. 12, or [34], p. 475). For *k* ∈ *s*, let *R*_*k*_ = 1 if *k* ∈ *r* and *R*_*k*_ = 0 otherwise. Under MCAR mechanism we have:

$$\begin{array}{c} \mathrm{Pr}[R_{k}=1]=\phi \;\text{for}\;\text{all}\;k\in s \end{array}$$ (5)

with 0 < *ϕ* < 1 the response propensity. Thus, as we suppose that the hunters respond independently from each other, the MCAR mechanism is a Bernoulli sampling (see [35], Section 3.2). It follows that, conditionally on *n_r_*, the sample *r* results from the application of a SRSWOR(*n*_*s*_, *n*_*r*_) (see [36], p. 44), or equivalently a SRSWOR(*N*, *n*_*r*_) (see [25], Theorem 4.1, p. 69). Thereby, under the MCAR mechanism of missingness, ${\bar{y}}_{r}$ is an unbiased estimator of $\bar{y}$.

A self-administered questionnaire can be paper- or web-based. A mail survey is potentially the most useful and inexpensive technique (by respect to interview-based surveys), and does not require access to the web nor computer skills, two things unequally shared by hunters between countries (and also within the same country). Accordingly, in what follows we will refer to mail surveys only.

In hunting bag mail surveys, the causes for nonresponse are partly common to any other type of mail survey: questionnaire never received, lost questionnaire, negligence, lack of time available, lack of interest. All these causes are not necessarily related to the hunting bag, hence several of these may be viewed as MCAR. For instance, nondelivery of the questionnaire is typically treated as an ignorable nonresponse cause [37, 38]. On the other hand, mail questionnaires are answered more often by people who, due to their educational and occupational background, more easily express themselves in writing. Writing facility is roughly correlated with educational level or socioeconomic status [39]. It cannot be taken for granted that this factor for responding is not related to the hunting bag. Another source of potential bias, at least at the regional scale, is related to the auspices, a conscious or unconscious slanting of responses because of attitudes toward the agency or organization sponsoring the survey [40]. This is for example a cause of bias that we can perfectly imagine in the case of France, where the hunters may behave differently towards various stakeholders within the hunting community (e.g. hunting NGOs versus national body). The demographic status of a certain game species may also influence the response. For instance, if the species is declining, some hunters may be afraid of publicizing their hunting bags just because they do not want to give clues to restrict their hunting activities even further. Anyway, there is a widespread nonresponse cause which is specific to hunting bag surveys, namely the tendency for nonrespondents to be less active or less successful hunters than are respondents [10, 13, 37, 38, 41, 42] (see also [43], Figure 6). Being related to the hunting bag, this nonresponse cause alone precludes ignoring the nonresponse as a source of (upward) bias. This is a well-documented and cogent argument that will be put at the heart of the present study.

### 1.5 Multiphase sampling approach

In this paper we only deal with sampling and nonresponse errors (we do not consider response and coverage errors). Several techniques for handling nonresponse problems in sample surveys are available in the literature. It is out of the scope of the present paper to review them in detail and the reader is referred to [44] (Chapter 8), [35] (Chapter 15), and [31, 45–47]. Basically, we may distinguish between, (i) methods applied at the design stage by ensuring that a subsample of the nonrespondents is followed up—a method pioneered by Hansen & Hurwitz [48]—and (ii), those applied at the estimation stage. These two types of methods can be combined as in [49] or [50]. All techniques in category (ii) use auxiliary information related to the variable of interest, or to the response propensity, in one way or another. For instance, if we had such variables for post-stratifying the sampling frame in strata homogeneous with respect to the hunting bag, or with respect to the propensity to respond, then the nonresponse bias could be greatly attenuated. Unfortunately, most of the time, relevant auxiliary information—in the sense that we have just specified—are not available in the context of hunting bag surveys. The mailing address, age and sex of the hunters usually available in the sampling frame are not such as allowing nonresponse bias attenuation, because they are not enough to give account for hunting bag or for response propensity. In principle, an auxiliary variable which could be very useful at the estimation stage would be the number of ammunitions fired during the hunting season under consideration. Indeed, such an information would allow identifying the least active hunters in the sample, who more likely had a null harvest. We could use this information for reweighting the respondents whose hunting bag was zero, and thus compensate for the deficit of null harvests among them. In practice, it is very unlikely to be able to gather relevant information about nonrespondents without contacting them. Accordingly, methods relying on auxiliary variables are generally not in use in our context (but see [51] for an example of imputation). Moreover, these methods may need assumptions which are difficult or impossible to verify. Lastly, when the hunting bag survey deals with a great number of game species (for instance, about 90 species in France), it is inconceivable to deal with the problem of nonresponse bias separately for each species. Therefore, we argue that the most practical solution in our context is design-based. Indeed, with a design-based approach, we avoid relying on uncheckable assumptions, and we are not limited in practice by the number of game species.

The first aim of this paper is to gather statistical elements scattered through the literature, and secondly to provide an unbiased estimator for the sampling variance (for any number of phases) which, to our knowledge, is still lacking. Although we consider nonrespondent subsampling designs because they are free from assumptions and do not require auxiliary variables, a practical requirement of major importance remains. Actually, the total estimator is unbiased only if the response rate at the last phase of the sampling design is 100%. The same holds for the sampling variance estimator. It is obvious that, in practice, the response will never reach 100% at the last phase (it was for instance only 75% in [37]), and theoretically the nonresponse bias issue hence remains [49]. Therefore, after describing the theory related to the sampling strategy that we advocate in this paper, the question still is whether or not the estimators are practically useful when some nonresponse remains at the last phase. In addition, it is necessary to provide some indications about the threshold response rate at the last phase under which the whole sampling strategy becomes useless, according to circumstances. To document this topic of utmost practical importance, we rely on Monte Carlo simulations. For this, we propose a nonresponse mechanism generating upward bias, which rely on the essential source of nonresponse error, namely the propensity of nonrespondents to have, on average, a lower hunting bag than respondents.

## Two-phase sampling design

We begin with the simplest case, which corresponds to the pioneering work of Hansen & Hurwitz [48]. Informally, their technique is applied as follows: (i) select a sample of hunters and mail a questionnaire to all of them, (ii) after the deadline has passed, identify the nonrespondents and select a subsample among them, (iii) collect the bags from the nonrespondents in the subsample by personal interview and (iv) combine data from the two sets of respondents for estimating the total hunting bag.

### 2.1 Design

Let *s*_1_ be the first-phase sample of size $n_{s_{1}}$ drawn from *U* by SRSWOR with sampling fraction $\nu =n_{s_{1}}/N$. A self-administered questionnaire is mailed to each surveyed person *k* ∈ *s*_1_. After the deadline to reply, the sample *s*_1_ can be partitioned into a subset of respondents *r*_1_ of size $n_{r_{1}}$, and a subset of nonrespondents *m*_1_ of size $n_{m_{1}}=n_{s_{1}}-n_{r_{1}}$. In the second phase, *m*_1_ is sampled by SRSWOR to obtain a subsample *s*_2_ of $n_{s_{2}}=n_{m_{1}}\nu_{m}$ (0 < *ν*_*m*_ ≤ 1) persons interviewed in face-to-face mode or by phone. In this phase, the response rate is assumed to be 100% ($n_{r_{2}}=n_{s_{2}}$). The design can thus be summarized by the scheme:

$$\begin{array}{c} \begin{array}{ccccccccc} U & \overset{\text{SRSWOR}}{⟶} & s_{1} & \to & r_{1} & & & & \\ & & & ↘ & & & & & \\ & & & & m_{1} & \overset{\text{SRSWOR}}{⟶} & s_{2} & = & r_{2} \end{array} \end{array}$$ (6)

Conditionally to the nonresponse, *U* may be viewed as poststratified into a strata of respondents *R* of size *N*_*R*_, with weight *W*_*R*_ = *N*_*R*_/*N*, and a strata of nonrespondents *M* of size *N*_*M*_, with weight *W*_*M*_ = *N*_*M*_/*N* = 1 − *W*_*R*_. Denoting ${\bar{y}}_{R}$ and ${\bar{y}}_{M}$ the mean in stratum *R* and *M*, respectively, the nonresponse bias can also be written as:

$$\begin{array}{c} \mathrm{NRBias}({\bar{y}}_{r})=E(N_{M}/N({\bar{y}}_{R}-{\bar{y}}_{M})) \end{array}$$ (7)

### 2.2 Mean and total estimators

The mean $\bar{y}$ in the population can be written as the linear combination:

$$\begin{array}{c} \bar{y}=W_{R}{\bar{y}}_{R}+W_{M}{\bar{y}}_{M} \end{array}$$ (8)

The sample *s*_1_ allows estimating *W*_*R*_ without bias by $w_{r}=n_{r_{1}}/n_{s_{1}}$. Similarly, *W*_*M*_ is estimated without bias by $w_{m}=n_{m_{1}}/n_{s_{1}}$. Unbiased mean and total estimators are, respectively:

$$\begin{array}{c} \begin{array}{cc} {\bar{y}}_{\text{HH}} & =w_{r}{\bar{y}}_{r_{1}}+w_{m}{\bar{y}}_{r_{2}}\\ & =\frac{1}{n_{s_{1}}}(n_{r_{1}}{\bar{y}}_{r_{1}}+n_{m_{1}}{\bar{y}}_{r_{2}}) \end{array} \end{array}$$ (9)

$$\begin{array}{c} \begin{array}{cc} {\hat{t}}_{\text{HH}} & =N\;{\bar{y}}_{\text{HH}}\\ & =\frac{N}{n_{s_{1}}}(n_{r_{1}}{\bar{y}}_{r_{1}}+n_{m_{1}}{\bar{y}}_{r_{2}}) \end{array} \end{array}$$ (10)

with ${\bar{y}}_{r_{1}}=n_{r_{1}}^{-1}\sum_{k\in r_{1}}y_{k}$ and ${\bar{y}}_{r_{2}}=n_{r_{2}}^{-1}\sum_{k\in r_{2}}y_{k}$. Estimators (9) and (10) are unbiased only when the response rate at the second phase is 100%.

### 2.3 Sampling variance

The sampling variance of ${\bar{y}}_{\text{HH}}$ may be written as:

$$\begin{array}{c} V_{p}({\bar{y}}_{\text{HH}})=(\frac{1}{n_{s_{1}}}-\frac{1}{N})S^{2}+\frac{1}{n_{s_{1}}}(\frac{1}{\nu_{m}}-1)W_{M}S_{M}^{2} \end{array}$$ (11)

with $S^{2}={\left(N-1\right)}^{-1}\sum_{k\in U}{\left(y_{k}-\bar{y}\right)}^{2}$ and $S_{M}^{2}={\left(N_{M}-1\right)}^{-1}\sum_{k\in M}{\left(y_{k}-{\bar{y}}_{M}\right)}^{2}$.

The first term corresponds to the first-phase SRSWOR variance, whereas the second term corresponds to the variance due to subsampling (second phase).

Note that the expression given by Hansen & Hurwitz [48] (Equation 2) involves “1/*N*” variances and not “1/(*N* − 1)” variances according to the current convention in the field of finite sampling theory (see for instance [52], p. 23). A demonstration of the variance expression is given by Hansen & Hurwitz [48] (Appendix), but also in [53] (p. 977) or [54] (pp. 204-205). Note also that the expression printed in [44] (p. 178, Equation 8.6) is erroneous because of the factorisation of the finite population correction.

The adaptation of two-phase sampling in the context of nonresponse leads to a specific instance of two-phase sampling for stratification (see [52], p. 371, or [55]). The theory for this latter design may be found in [56], [52] (pp. 327-335), [57] (pp. 90-92) or [58]. In the second phase, the stratified sample on which the estimation is based is composed of:

1. the totality of *r*_1_, that is subsampling is performed by taking *ν*_*r*_ = 1 (exhaustive “subsampling”),
2. a subsample *s*_2_ drawn from *m*_1_ by SRSWOR, with 0 < *ν*_*m*_ ≤ 1.

### 2.4 Sampling variance estimator

A sampling variance estimator was not given by Hansen & Hurwitz [48]. To obtain a nonnegative unbiased variance estimator, just start for instance from the formula of the variance estimator given by Rao [56]. After some algebraic simplifications we obtain:

$$\begin{array}{c} \begin{array}{cc} {\hat{V}}_{p}({\bar{y}}_{\text{HH}})= & \frac{\left(n_{r_{1}}-1\right)\left(N-n_{s_{1}}\right)}{N\left(n_{s_{1}}-1\right)n_{s_{1}}}S_{r_{1}}^{2}+\frac{N-1}{N}(\frac{n_{m_{1}}-1}{n_{s_{1}}-1}-\frac{n_{r_{2}}-1}{N-1})\frac{w_{m_{1}}}{n_{r_{2}}}S_{r_{2}}^{2}\\ & +\frac{N-n_{s_{1}}}{N(n_{s_{1}}-1)}[w_{r_{1}}{\left({\bar{y}}_{r_{1}}-{\bar{y}}_{\text{HH}}\right)}^{2}+w_{m_{1}}{\left({\bar{y}}_{r_{2}}-{\bar{y}}_{\text{HH}}\right)}^{2}] \end{array} \end{array}$$ (12)

with $S_{r_{1}}^{2}={\left(n_{r_{1}}-1\right)}^{-1}\sum_{k\in r_{1}}{\left(y_{k}-{\bar{y}}_{r_{1}}\right)}^{2}$ and $S_{r_{2}}^{2}={\left(n_{r_{2}}-1\right)}^{-1}\sum_{k\in r_{2}}{\left(y_{k}-{\bar{y}}_{r_{2}}\right)}^{2}$.

Expression (12) is algebraically equivalent to those provided in [59] (Equation 11), [60] (p. 304), [61] (p. 332, Equation 13.5), and [62] (Equation 9) or [34] (p. 473). Another expression is given in S1 Appendix, in line with our generalized estimator for any number of phases (see next section). Lohr [63] (p. 338) also provides a simplified expression which assumes the finite population corrections can be neglected.

## Multiphase sampling for nonresponse

El-Badry [64] generalized the method of Hansen & Hurwitz [48] to any number *ℓ* of mailing waves, followed by a last phase *L* = *ℓ* + 1 for personal interview. The latter phase has a supposed response rate of 100%.

In extending the two-phase case, now the population *U* is stratified into *ℓ* strata *R*_*i*_ containing $N_{R_{i}}$ persons who respond to the *i*-th mailing wave, plus a strata *R*_*L*_ with $N_{R_{L}}$ persons who not yet responded after *ℓ* mailing waves but are assumed to respond to an interviewer, in face-to-face mode or by phone.

To each stratum *R*_*i*_ with weight $W_{R_{i}}=N_{R_{i}}/N$ is associated a nonrespondent strata *M*_*i*_. Letting *M*_0_ = *U* and *R*_0_ = ∅, the partition of *U* (for 1 ≤ *i* ≤ *ℓ*) may be written as:

$$\begin{array}{c} U=\overset{i-1}{\underset{j=0}{\cup }}R_{j}\cup R_{i}\cup \underset{M_{i}}{\underset{︸}{\overset{L}{\underset{j=i+1}{\cup }}R_{j}}} \end{array}$$ (13)

with, in particular, *M*_*ℓ*_ = *R*_*L*_. For instance, for *L* = 5 (*ℓ* = 4) we get the scheme:

$$\begin{array}{c} \begin{array}{ccccccccccc} U=M_{0} & \to & R_{1} & & & & & & & & \\ & ↘ & & & & & & & & & \\ & & M_{1} & \to & R_{2} & & & & & & \\ & & & ↘ & & & & & & & \\ & & & & M_{2} & \to & R_{3} & & & & \\ & & & & & ↘ & & & & & \\ & & & & & & M_{3} & \to & R_{4} & & \\ & & & & & & & ↘ & & & \\ & & & & & & & & M_{4} & \to & R_{5} \end{array} \end{array}$$ (14)

With $W_{M_{\ell }}=W_{R_{L}}$, the weights $W_{M_{i}}$ (0 ≤ *i* < *ℓ*) are defined by the recurrence relation $W_{M_{i}}=W_{R_{i+1}}+W_{M_{i+1}}$.

### 3.1 Design

Considering *ℓ* mailing waves, the design is the following:

- the first mailing wave (*i* = 1) is an SRSWOR from population *U*,
- if *ℓ* > 1, each following mailing wave 1 < *i* ≤ *ℓ* is addressed to a subsample drawn by SRSWOR from the nonrespondents of the previous mailing wave (*i* − 1),
- the last subsample drawn by SRSWOR (*i* = *ℓ* + 1 = *L*) concerns the nonrespondents of the wave *ℓ* to whom we resort to personal interview for ensuring a 100% response rate.

For instance, for *L* = 3 (*ℓ* = 2) we have the scheme:

$$\begin{array}{c} \begin{array}{ccccccccccccc} U & \overset{\text{SRSWOR}}{⟶} & s_{1} & \to & r_{1} & & & & & & & & \\ & & & ↘ & & & & & & & & & \\ & & & & m_{1} & \overset{\text{SRSWOR}}{⟶} & s_{2} & \to & r_{2} & & & & \\ & & & & & & & ↘ & & & & & \\ & & & & & & & & m_{2} & \overset{\text{SRSWOR}}{⟶} & s_{3} & = & r_{3} \end{array} \end{array}$$ (15)

Letting $n_{m_{0}}=N$, 0 < *ν*_*i*_ ≤ 1, the size of each successive sample *s*_*i*_ is defined as $n_{s_{i}}=n_{m_{i-1}}\nu_{i}$, and therefore $E_{p}\left(n_{s_{i}}\right)=\nu_{i}E_{p}\left(n_{m_{i-1}}\right)$, for 1 ≤ *i* ≤ *L*.

### 3.2 Mean and total estimators

The population mean can be written as a linear combination of the respondent strata means:

$$\begin{array}{c} \bar{y}=\sum_{i=1}^{L}W_{R_{i}}\;{\bar{y}}_{R_{i}} \end{array}$$ (16)

We have (see for instance [65], p. 122):

$$\begin{array}{cc} & E_{p}(\frac{n_{r_{1}}}{n_{s_{1}}})=E_{p}(\frac{n_{r_{1}}}{N\nu_{1}})=W_{R_{1}} \end{array}$$ (17)

$$\begin{array}{cc} & E_{p}(\frac{n_{r_{2}}}{n_{s_{1}}\nu_{2}})=E_{p}(\frac{n_{r_{2}}}{N\nu_{1}\nu_{2}})=W_{R_{2}} \end{array}$$ (18)

$$\begin{array}{c} {\text{E}}_{p}(\frac{n_{r_{3}}}{n_{s_{1}}\nu_{2}\nu_{3}})=E_{p}(\frac{n_{r_{3}}}{N\nu_{1}\nu_{2}\nu_{3}})=W_{R_{3}}\\ \vdots \end{array}$$ (19)

$$\begin{array}{cc} & E_{p}(n_{r_{\ell }}/n_{s_{1}}\prod_{j=2}^{\ell }\nu_{j})=E_{p}(n_{r_{\ell }}/N\prod_{j=1}^{\ell }\nu_{j})=W_{R_{\ell }} \end{array}$$ (20)

Letting $\Pi_{i}=\prod_{j=1}^{i}\nu_{j}$ for 1 ≤ *i* ≤ *ℓ*, we obtain the general term:

$$\begin{array}{c} E_{p}(\frac{n_{r_{i}}}{N\Pi_{i}})=W_{R_{i}} \end{array}$$ (21)

Accordingly, we have the unbiased estimators (1 ≤ *i* ≤ *ℓ*):

$$\begin{array}{c} {\hat{W}}_{R_{i}}=\frac{n_{r_{i}}}{N\Pi_{i}} \end{array}$$ (22)

and for *i* = *L* we get:

$$\begin{array}{c} E_{p}(\frac{n_{m_{\ell }}}{N\Pi_{\ell }})=W_{R_{L}} \end{array}$$ (23)

which leads to the unbiased estimator:

$$\begin{array}{c} {\hat{W}}_{R_{L}}=\frac{n_{m_{\ell }}}{N\Pi_{\ell }} \end{array}$$ (24)

The mean $\bar{y}$ can be estimated without bias using ${\bar{y}}_{\text{EB}}$ (e.g. [64], Equation 3), which can be written with our notations as:

$$\begin{array}{c} \begin{array}{cc} {\bar{y}}_{\text{EB}} & =\sum_{i=1}^{L}{\hat{W}}_{R_{i}}\;{\bar{y}}_{r_{i}}\\ & =\sum_{i=1}^{\ell }\frac{n_{r_{i}}}{N\Pi_{i}}{\bar{y}}_{r_{i}}+\frac{n_{m_{\ell }}}{N\Pi_{\ell }}{\bar{y}}_{r_{L}}\\ & =\frac{1}{N}(\sum_{i=1}^{\ell }\frac{n_{r_{i}}}{\Pi_{i}}{\bar{y}}_{r_{i}}+\frac{n_{m_{\ell }}}{\Pi_{\ell }}{\bar{y}}_{r_{L}}) \end{array} \end{array}$$ (25)

Of course, the total estimator is:

$$\begin{array}{c} \begin{array}{cc} {\hat{t}}_{\text{EB}} & =N\;{\bar{y}}_{\text{EB}}\\ & =\sum_{i=1}^{\ell }\frac{n_{r_{i}}}{\Pi_{i}}{\bar{y}}_{r_{i}}+\frac{n_{m_{\ell }}}{\Pi_{\ell }}{\bar{y}}_{r_{L}} \end{array} \end{array}$$ (26)

In practice, due to the rounding necessary to obtain integer sample sizes, in place of the sampling fractions provided by the design, we prefer to write the estimator by explicitly showing the sample sizes used:

$$\begin{array}{c} {\hat{t}}_{\text{EB}}=\sum_{i=1}^{\ell }\frac{n_{r_{i}}}{\prod_{j=1}^{i}\frac{n_{s_{j}}}{n_{m_{j-1}}}}{\bar{y}}_{r_{i}}+\frac{n_{m_{\ell }}}{\prod_{j=1}^{\ell }\frac{n_{s_{j}}}{n_{m_{j-1}}}}{\bar{y}}_{r_{L}} \end{array}$$ (27)

To ensure, on the average, the sampling fractions provided by the design, it is necessary that the sampling sizes $n_{s_{i}}$ be rounded by randomizing between $\left\lfloor n_{s_{i}}\right\rfloor$ and $\left\lceil n_{s_{i}}\right\rceil$ with respective probabilities $1-n_{s_{i}}+\left\lfloor n_{s_{i}}\right\rfloor$ and $n_{s_{i}}-\left\lfloor n_{s_{i}}\right\rfloor$ [66]. This point is important in case of Monte Carlo simulation (see Section 4.2.1).

Again, estimators (25) and (26) are unbiased if response rate is really 100% at the last phase. For *L* = 2, the estimator ${\bar{y}}_{\text{HH}}$ (9) is obtained as a particular instance of the estimator ${\bar{y}}_{\text{EB}}$ (25). Taking *L* = 3, we obtain:

$$\begin{array}{c} {\bar{y}}_{\text{EB}}=\frac{1}{n_{s_{1}}}(n_{r_{1}}{\bar{y}}_{r_{1}}+\frac{n_{r_{2}}}{\nu_{2}}{\bar{y}}_{r_{2}}+\frac{n_{m_{2}}}{\nu_{2}}{\bar{y}}_{r_{3}}) \end{array}$$ (28)

in accordance with the expression given by Siripornpibul [67] (p. 66, Equation 3.1), but with a different notation.

### 3.3 Sampling variance

The sampling variance for ${\bar{y}}_{\text{EB}}$ was given by El-Badry [64] (Equation 4) (see also [68], pp. 407-409). As Rao [62] (p. 105, Equation 36), we prefer the variance expression given by Srinath [69] (Equation 2.16), that is, with our notations:

$$\begin{array}{c} \begin{array}{cc} V_{p}({\bar{y}}_{\text{EB}})= & (\frac{1}{n_{s_{1}}}-\frac{1}{N})S^{2}+\frac{1}{n_{s_{1}}}(\frac{1}{\nu_{2}}-1)W_{M_{1}}S_{M_{1}}^{2}\\ & +\frac{1}{n_{s_{1}}}\sum_{i=2}^{\ell }(\prod_{j=2}^{i}\frac{1}{\nu_{j}})(\frac{1}{\nu_{i+1}}-1)W_{M_{i}}S_{M_{i}}^{2} \end{array} \end{array}$$ (29)

with $S_{M_{i}}^{2}={\left(N_{M_{i}}-1\right)}^{-1}\sum_{k\in M_{i}}{\left(y_{k}-{\bar{y}}_{M_{i}}\right)}^{2}$. For *L* = 2, the third term in (29) is not defined and we obtain the variance (11) as a special case. For *L* = 3, we obtain:

$$\begin{array}{cc} V_{p}({\bar{y}}_{\text{EB}})= & (\frac{1}{n_{s_{1}}}-\frac{1}{N})S^{2}+\frac{1}{n_{s_{1}}}(\frac{1}{\nu_{2}}-1)W_{M_{1}}S_{M_{1}}^{2} \end{array}$$ (30)

$$\begin{array}{cc} & +\frac{1}{n_{s_{1}}}\frac{1}{\nu_{2}}(\frac{1}{\nu_{3}}-1)W_{M_{2}}S_{M_{2}}^{2} \end{array}$$ (31)

in accordance with the expression given by Siripornpibul [67] (p. 66, Equation 3.2), but with a different notation.

Letting:

$$\begin{array}{c} \Pi_{i}=\{\begin{array}{cc} 1 & \;\text{if}\;i=1\\ \prod_{j=1}^{i-1}\frac{1}{\nu_{j}} & \;\text{if}\;i>1 \end{array} \end{array}$$

the variance (29) can be rewritten in a more compact way as:

$$\begin{array}{c} V_{p}({\bar{y}}_{\text{EB}})=\frac{1}{N}\sum_{i=1}^{L}\Pi_{i}(\frac{1}{\nu_{i}}-1)W_{M_{i-1}}S_{M_{i-1}}^{2} \end{array}$$ (32)

and likewise the variance for the total estimator can be written as:

$$\begin{array}{c} \begin{array}{cc} V_{p}({\hat{t}}_{\text{EB}}) & =N^{2}V_{p}({\bar{y}}_{\text{EB}})\\ & =\sum_{i=1}^{L}\Pi_{i}(\frac{1}{\nu_{i}}-1)N_{M_{i-1}}S_{M_{i-1}}^{2} \end{array} \end{array}$$ (33)

### 3.4 Sampling variance estimator

Again, a sampling variance estimator was not given by El-Badry [64]. After generalizing the sampling variance estimator for multiphase sampling for stratification to any number of phases (for two-, three-, and four-phase sampling for stratification, see [57], pp. 81-118, and [58]), and after some algebraic simplifications, we obtain the general expression:

$$\begin{array}{c} \begin{array}{cc} {\hat{V}}_{p}({\hat{t}}_{\text{EB}})= & \sum_{i=1}^{\ell }\Pi_{i}[\frac{n_{m_{i-1}}\left(n_{m_{i-1}}-n_{s_{i}}\right)}{n_{s_{i}}\left(n_{s_{i}}-1\right)}](z_{i}-\frac{1}{n_{s_{i}}}t_{i}^{2})+\\ & \Pi_{L}[n_{m_{\ell }}^{2}(\frac{1}{n_{s_{L}}}-\frac{1}{n_{m_{\ell }}})S_{r_{L}}^{2}] \end{array} \end{array}$$ (34)

with:

$$\begin{array}{c} \Pi_{i}=\{\begin{array}{cc} 1\; & \text{if}\;i=1\\ \prod_{j=1}^{i-1}\frac{n_{m_{j-1}}\left(n_{m_{j-1}}-1\right)}{n_{s_{j}}\left(n_{s_{j}}-1\right)}\; & \text{if}\;i>1 \end{array} \end{array}$$

and for 1 ≤ *i* ≤ *ℓ*:

$$\begin{array}{cc} t_{L} & =\sum_{k\in r_{L}}y_{k} \end{array}$$ (35)

$$\begin{array}{cc} t_{i} & =\sum_{k\in r_{i}}y_{k}+\frac{n_{m_{i}}}{n_{s_{i+1}}}t_{i+1} \end{array}$$ (36)

$$\begin{array}{cc} z_{L} & =\sum_{k\in r_{L}}y_{k}^{2} \end{array}$$ (37)

$$\begin{array}{cc} z_{i} & =\sum_{k\in r_{i}}y_{k}^{2}+\frac{n_{m_{i}}}{n_{s_{i+1}}}z_{i+1} \end{array}$$ (38)

For *L* = 2 and *L* = 3 we obtain as particular instances the sampling variance estimators given in S1 Appendix.

## Simulating the nonresponse bias

Although the nonresponse bias elimination strategy we presented (through multiphase sampling) is not restricted to hunting bag surveys, the nonresponse mechanism we propose in this section is very specific to the matter at hand.

### 4.1 Nonresponse mechanism

We separate ignorable causes of nonresponse from nonignorable ones (i.e. related to values taken by *y*). For the sake of simplicity, among the nonrespondents, we consider the propensity to not respond when the hunting bag is zero (nonactive hunter or unsuccessful hunter) as the only cause of nonignorable nonresponse.

Within *U* we distinguish the stratum *U*_0_ ∋ *k* such as *y*_*k*_ = 0, from the stratum *U*_1_ ∋ *k* such as *y*_*k*_ > 0. A sample *s* of size *n_s_* is drawn by SRSWOR from *U*. We define *s*_0_ = *s* ∩ *U*_0_ of size *n*_0_ and *s*_1_ = *s* ∩ *U*_1_ of size *n*_1_. The set-size *n*_0_ is an outcome of a random variable because of the replication of the random draw by SRSWOR. This size follows a hypergeometric distribution whose probability mass function (pmf) is:

PH(n0|ns,N0,N)={(N0n0)(N-N0ns-n0)(Nns)-1ifn0∈DH(ns,N0,N)0otherwise (39)

with $N\in N^{*}$, $n_{s}\in {\mathbb{N}}^{*}$, $N_{0}\in N^{*}$ and domain $D_{\text{H}}\left(n_{s},N_{0},N\right)=\left(\max\left(0,n_{s}+N_{0}-N\right),\cdots ,\min\left(n_{s},N_{0}\right)\right)$ [70] (p. 251). The mean and variance of *n*_0_ are, respectively:

$$\begin{array}{c} E_{\text{H}}(n_{0})=n_{s}\frac{N_{0}}{N}=n_{s}W_{0} \end{array}$$ (40)

$$\begin{array}{c} \begin{array}{cc} V_{\text{H}}(n_{0}) & =n_{s}\frac{N_{0}}{N}(1-\frac{N_{0}}{N})\frac{N-n_{s}}{N-1}\\ & =n_{s}W_{0}\left(1-W_{0}\right)\frac{N-n_{s}}{N-1} \end{array} \end{array}$$ (41)

Conditionally to the sample *s* (and thus to *n*_0_ and *n*_1_), the nonresponse can be viewed as a second sampling phase. Let 0 ≤ *π*_*m*_ < 1 the propensity to nonrespond, all causes of nonresponse confounded, and let 0 ≤ *π*_*z*_ ≤ 1 the propensity, among the nonrespondents, to nonrespond because their hunting bag was zero. Let *z* be the size of *n_z_*, the set of nonrespondents who nonrespond because their harvest is null, with *n_z_* ≤ *n_m_* and *n_z_* ≤ *n*_0_. We have:

$$\begin{array}{cc} & E(n_{m})=n_{s}\pi_{m} \end{array}$$ (42)

$$\begin{array}{cc} & E(n_{z})=n_{s}\pi_{z}\pi_{m} \end{array}$$ (43)

The nonresponse bias can be written as:

$$\begin{array}{c} \mathrm{NRBias}({\bar{y}}_{r})=E(n_{z}/n_{s}\;{\bar{y}}_{r}) \end{array}$$ (44)

With *n*_*z*_ independent from ${\bar{y}}_{r}$, the nonresponse bias can also be written as:

$$\begin{array}{c} \mathrm{NRBias}({\bar{y}}_{r})=\pi_{z}\pi_{m}E({\bar{y}}_{r}) \end{array}$$ (45)

If *π*_*z*_ = 0 then *n_z_* = 0 (∀*n_m_*) and $\mathrm{NRBias}({\bar{y}}_{r})=0$ (the nonresponse is ignorable). Under the constraint *n_z_* ≤ *n*_0_, if *π*_*z*_ = 1 then *n_z_* = *n_m_* and $\mathrm{NRBias}({\bar{y}}_{r})$ is maximal for a given *π*_*m*_.

### 4.2 Simulating the nonresponse mechanism

We now describe the way we implement the nonresponse mechanism specific to hunting bag surveys. For uni-phase SRSWOR the sampled population is of course *U*. For the multiphase sampling strategy, the algorithm we propose is successively applied to *m*_*j*_ for *j* = 0, 1, …, *ℓ* (with *m*_0_ = *U*) for generating *s*_1_, *s*_2_, …, *s*_*L*_. For the sake of notation simplicity, in what follows we describe the algorithm when sampling *U*.

#### 4.2.1 Randomizing a set-size

In a Monte Carlo simulation of sampling, all set-sizes are necessarily integers. However, their expectations are not necessarily integers but must be approximately respected during the simulation. To randomly generate a set-size *n* such as E(*n*) = *Nπ* = *α*, with $N\in N^{*}$, 0 ≤ *π* ≤ 1, and n∈N, we used the two-point distribution:

$$\begin{array}{c} {\text{P}}_{\text{T}}\left(n|N,\pi \right)=\{\begin{array}{cc} \omega \; & \text{if}\;n=\lfloor \alpha \rfloor +1\\ 1-\omega \; & \text{if}\;n=\lfloor \alpha \rfloor \\ 0 & \text{otherwise} \end{array} \end{array}$$ (46)

with *ω* = *α* − ⌊*α*⌋, or equivalently:

$$\begin{array}{c} {\text{P}}_{\text{T}}\left(n|N,\pi \right)=\{\begin{array}{cc} \omega^{x}{\left(1-\omega \right)}^{1-x}\; & \text{if}\;n=\lfloor \alpha \rfloor +x\text{,}\;x\in \{0,1\}\\ 0 & \text{otherwise} \end{array} \end{array}$$ (47)

of mean E_T_(*n*) = *Nπ* and variance V_T_(*n*) = *ω*(1 − *ω*).

#### 4.2.2 Simulation algorithm

In the context of this article, the scheme we used to simulate the nonresponse mechanism consists of randomly defining a set of respondents *R* ⊂ *U* of size *N*_*R*_ and a set of nonrespondents *M* ⊂ *U* of size *N*_*M*_, with *U* = *R* ∪ *M* and *R* ∩ *M* = ∅. Within *M* we define the subset *Z* ⊂ *U*_0_ of size *N*_*Z*_ of hunters nonresponding because their hunting bag was zero. If *N*_*Z*_ > 0, then the null hunting bags are overrepresented within *M*, and there exists an upward nonresponse bias. The algorithm is the following:

1. randomly generate *N*_*M*_ such as E(*N*_*M*_) = *Nπ_m_*

2. *N*_*R*_ ← *N* − *N*_*M*_

3. randomly generate *N*_*Z*_ such as E(*N_Z_*) = *Nπ*_*z*_*π_m_*

4. a sample *Z* is drawn from *U*_0_ by SRSWOR(*N*_0_, *N_z_*)

5. *C* ← *U* − *Z*, *N*_*C*_ ← *N* − *N*_*Z*_

6. a sample *R* is drawn from *C* by SRSWOR(*N_C_*, *N_R_*)

7. *M* ← *U* − *R*, (*M* ⊃ *Z*)

8. a sample *s* is drawn from *U* by SRSWOR(*N*, *n_s_*)

9. *m* ← *s* ∩ *M*

10. *z* ← *s* ∩ *Z*

11. *r* ← *s* ∩ *R*

With *N*_*Z*_ independent from ${\bar{y}}_{R}$, the nonresponse bias can also be written as:

$$\begin{array}{c} \mathrm{NRBias}({\bar{y}}_{r})=\pi_{z}\pi_{m}E({\bar{y}}_{R}) \end{array}$$ (48)

**Fig 1:**
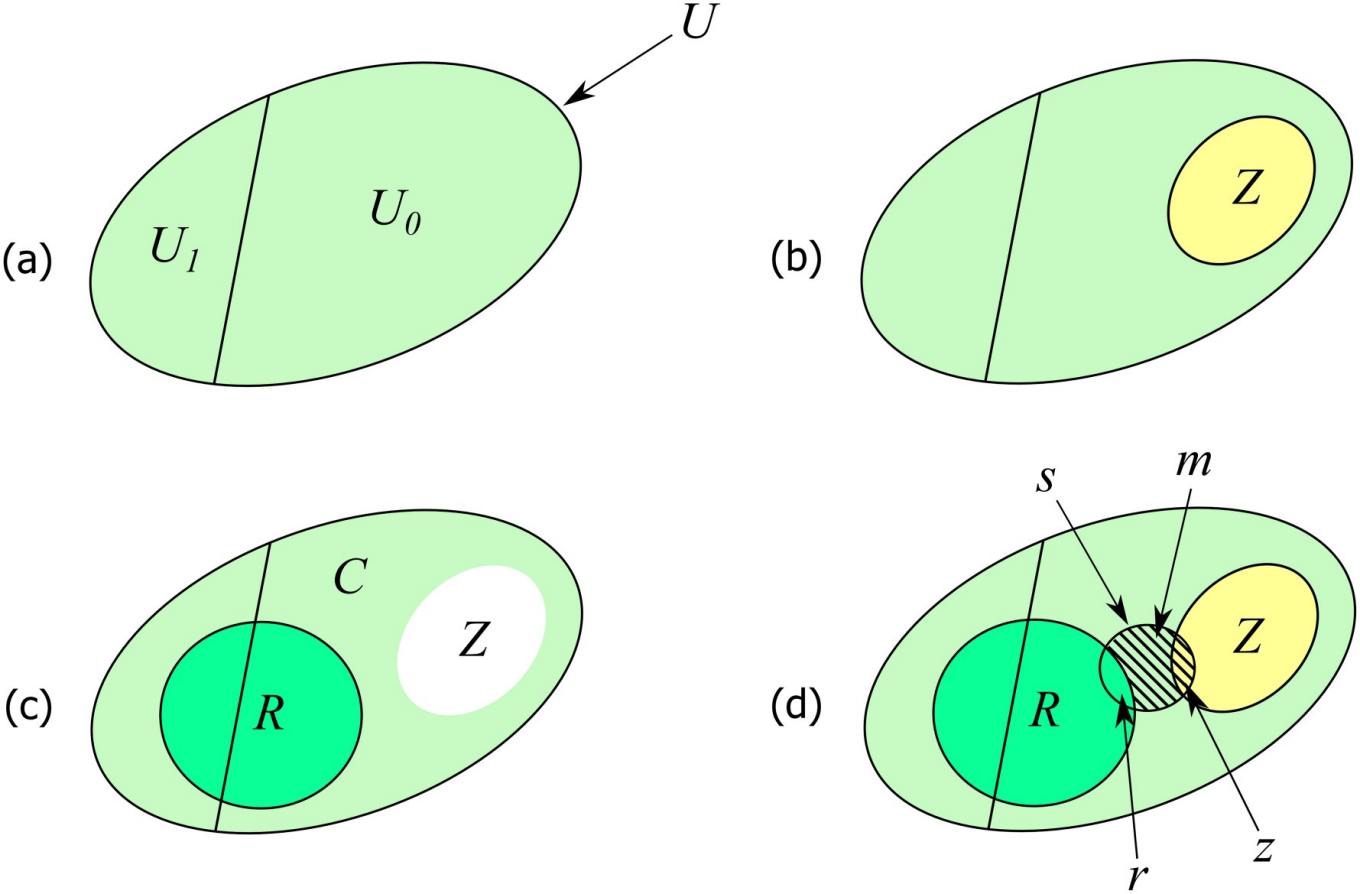
Scheme of the algorithm steps for implementing the nonresponse mechanism. (a) partition of *U* into strata *U*_0_ ∋ *k* such as *y*_*k*_ = 0 and *U*_1_ ∋ *k* such as *y*_*k*_ > 0; (b) step 4: a random subset *Z* is defined within *U*_0_; (c) steps 5-6: the “hole” in *U* corresponds to *Z*, resulting in an undercoverage of the stratum *U*_0_ when selecting the random set *R* within *C*: no elements which belong to *Z* can be included in *R*; (d) steps 7-11: random selection of the sample *s* within *U*: the random sets *r*, *m* (hatched area within *s*) and *z* result from the intersection of *s* with *R*, *M*, and *Z*, respectively (with *z* included within *m* since *Z* is included within *M*).

Under this algorithm, the distributional properties of *n_m_* and *n_z_* are given in S1 Appendix. At step 3 of the algorithm, it is required that two constraints are satisfied, namely *N*_*Z*_ ≤ *N*_*M*_ and *N*_*Z*_ ≤ *N*_0_. These constraints are also examined in S1 Appendix. For the reader convenience, Fig 1 illustrate the algorithm.

## Monte Carlo simulation study

According to the nonresponse mechanism we proposed in section 4.1 and the algorithm described in section 4.2.2, it is possible to vary the values of *π*_*m*_ and *π*_*z*_ at each phase. Besides, we do not want only theoretical results, but orders of magnitude rooted in reality. Consequently, since the multiphase sampling strategy is complex, and given the possibility to vary the nonresponse at each phase and the requirement of concrete results, we rely on Monte Carlo simulations to documentate the bias of the estimators. For ensuring the quality of our Monte Carlo simulations, we used several random number streams with huge period and very good properties by using function MRG32k3a proposed by L’Ecuyer [71] (Fig 1).

### 5.1 Superpopulation model

To simulate a set of individual hunting bags we need a superpopulation model *ξ* which should be a discrete distribution allowing to use any proportion of null values. To specify *ξ* for simulation purpose, a convenient choice is a two-parameter distribution such as the hurdle-at-zero Poisson model:

$$\begin{array}{c} \mathrm{Pr}(Y=y)=\{\begin{array}{cc} p & \;\text{if}\;y=0\\ \phi \frac{e^{-\lambda }\lambda^{y}}{y!} & \;\text{for}\;y=1,2,\ldots \end{array} \end{array}$$ (49)

with 0 ≤ *p* ≤ 1, *ϕ* = (1 − *p*)/(1 − *e*^−λ^) and *ϕ* ≤ (1 − *e*^−λ^)^−1^ [70] (p. 352). This distribution is over- or underdispersed by respect to the Poisson distribution depending on the value of *ϕ* ≠ 1. If *ϕ* = 1 then we have *p* = *e*^−λ^ and we obtain the Poisson distribution as a particular instance. Mean and variance are [70] (p. 352):

μ=ϕλ (50)

$$\mu_{2}=\phi \lambda \left(1+\lambda \right)-\phi^{2}\lambda^{2}$$ (51)

Knowing *μ*, we can obtain the value of parameter λ as a solution of the transcendental equation *μ* = λ(1 − *p*)/(1 − *e*^−λ^), that is:

$$\begin{array}{c} \lambda =W_{0}\left(De^{D}\right)-D \end{array}$$ (52)

with *D* = *μ*/(*p* − 1) and *W*_0_(*x*) ≥ −1 the upper branch of the Lambert function.

### 5.2 Nonresponse bias

When estimating a parameter *ω* using an estimator $\hat{\omega }$, we define the bias index $r=E(\hat{\omega })/\omega$. The bias $E\left(\hat{\omega }\right)-\omega$ is positive for *r* > 1, null for *r* = 1, and negative for *r* < 1. Here we plot the bias index $r=E({\bar{y}}_{r})/\bar{y}$.

We simulate one finite population *U* of size *N* = 10 000 by sampling the superpopulation model *ξ* with parameters *p* = 0.955 and λ = 7, that is for a superpopulation mean *μ* ≃ 0.315. For the population simulated we have *N*_0_ = 9534. We replicate 100 000 times the algorithm simulating the nonresponse mechanism with a sampling fraction *ν* = *n*/*N* = 0.5, for *π*_*m*_ = 0(0.05)0.9 and *π*_*z*_ = 0(0.05)0.9.

**Fig 2:**
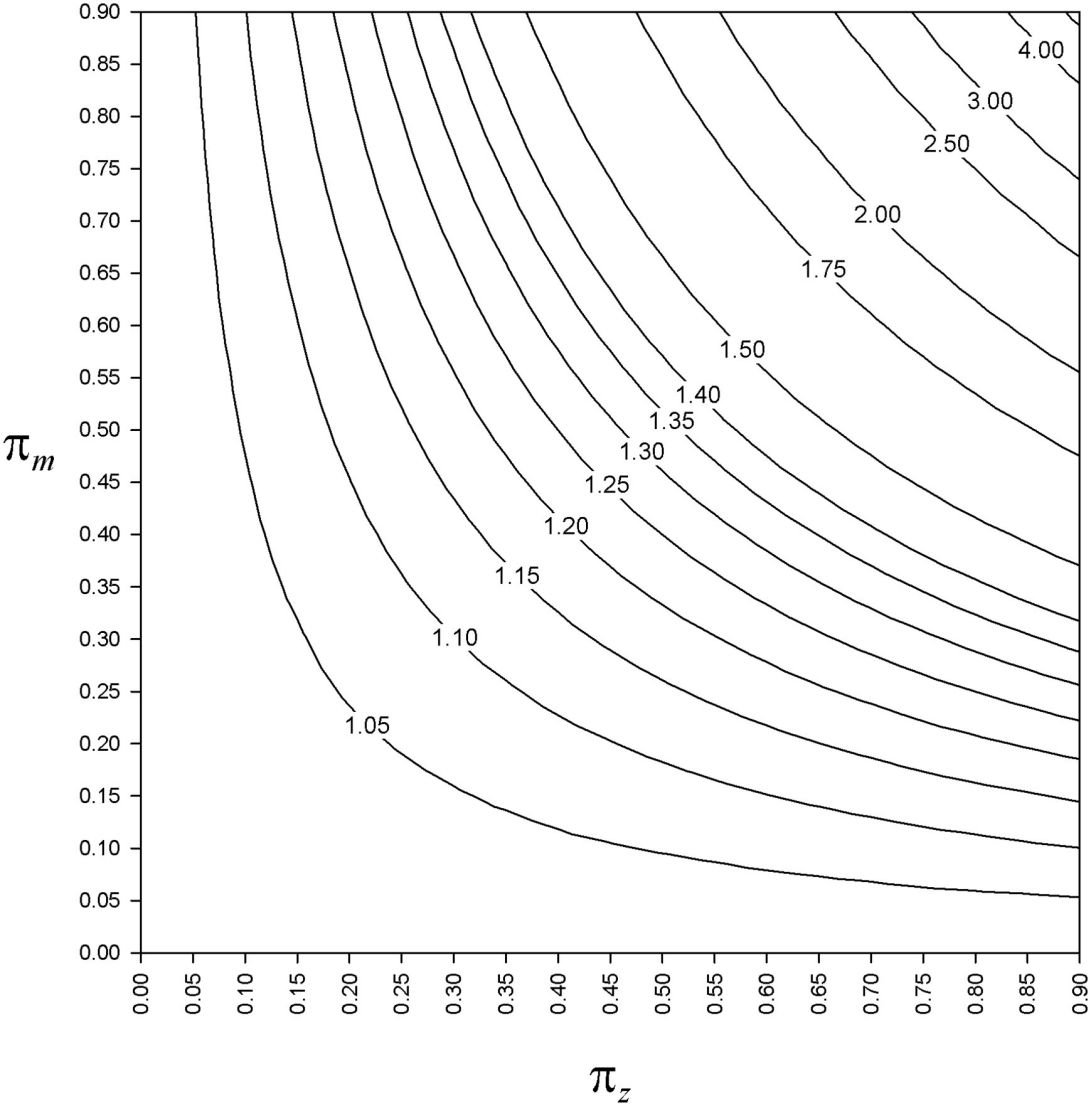
For the simulated population, bias index of the sample mean as a function of π_*m*_ and π_*z*_. Contour levels for the bias index $r=E({\bar{y}}_{r})/\bar{y}$ based on 100 000 simulations of the nonresponse mechanism, for *π*_*m*_ = 0(0.05)0.9 and *π*_*z*_ = 0(0.05)0.9. The contour level for *r* = 1 is confounded with the axes (*π*_*z*_ = 0 and *π*_*m*_ = 0). Details in the text.

In accordance with the nonresponse bias expression (45), Fig 2 shows that *π*_*z*_ and *π*_*m*_ play a symmetrical role in the magnitude of the nonresponse bias. Recall that the sampling fraction *ν* plays no role here. For instance, with *ν* = 0.05 we would get exactly the same figure (we should just increase the number of simulations to get such smooth contour levels as depicted here). For the finite population simulated, for *π*_*m*_ = 0.85 and *π*_*z*_ = 0.30, the classical estimator ${\bar{y}}_{r}$ leads to an overestimation of about 34%. Again for *π*_*m*_ = 0.85 but with *π*_*z*_ = 0.20, the overestimation is about 20%.

### 5.3 Nonresponse bias attenuation under two-phase design

**Fig 3:**
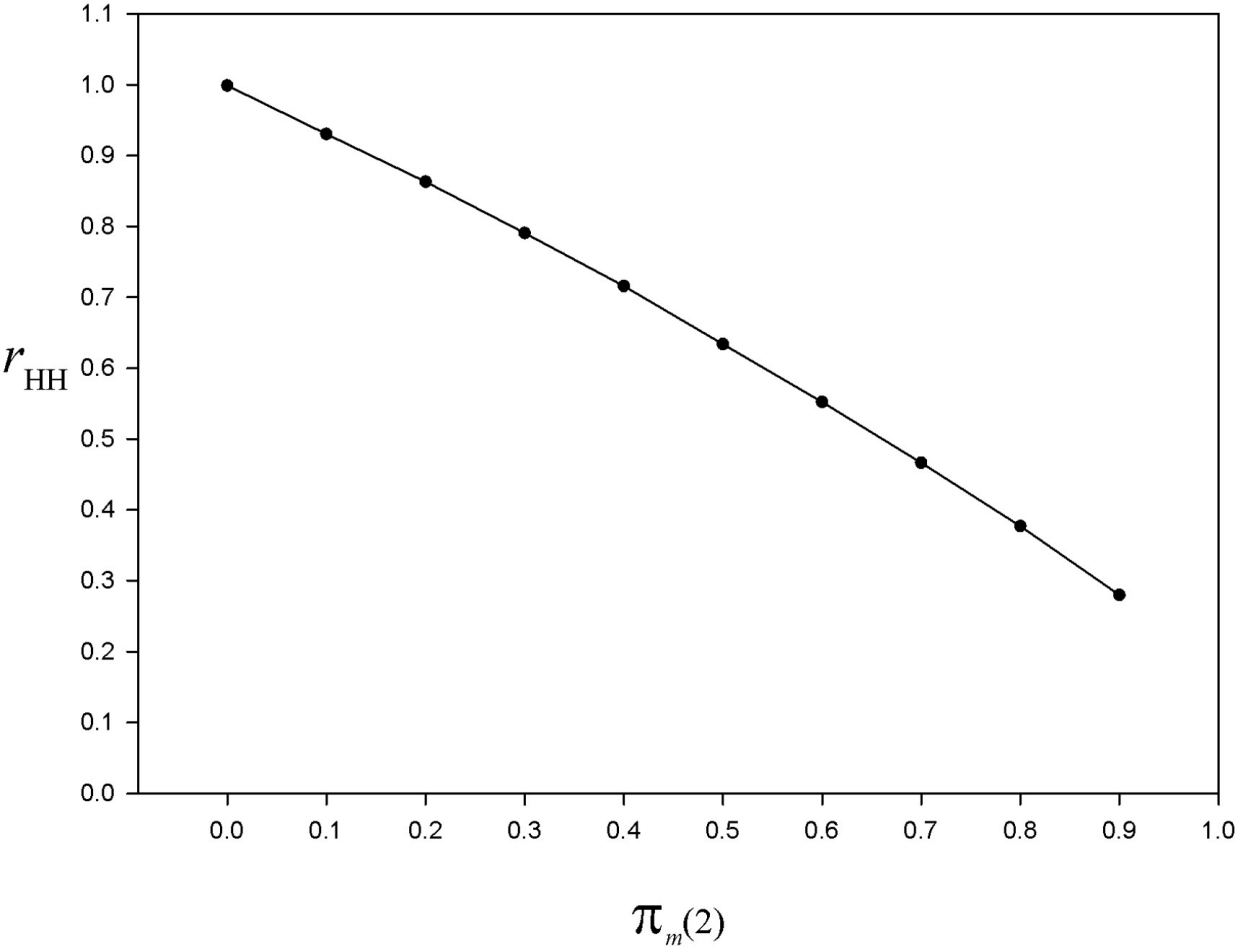
For the simulated population, bias index of the Hansen & Hurwitz estimator as a function of the nonresponse rate at the last phase. Curve of the bias index $r_{\text{HH}}=E({\bar{y}}_{\text{HH}})/\bar{y}$ based on 1000 simulations of the nonresponse mechanism, for *π*_*z*_ = 0.2, *π*_*m*_(1) = 0.85, and *π*_*m*_(2) = 0(0.1)0.9. Details in the text.

Let *π*_*m*_(1) and *π*_*m*_(2) the values of *π*_*m*_ at the first and second phase of the two-phase sampling design (section 2), respectively. Using the estimator ${\bar{y}}_{\text{HH}}$ (9), whatever the value of *π*_*m*_(1) < 1, for *π*_*m*_(2) = 0 the nonresponse bias is eliminated. When *π*_*m*_(2) > 0, then the nonresponse bias is only attenuated. For the same finite population as in section 5.2, under a two-phase sampling design with sampling fractions *ν* = *ν*_*m*_ = 0.5, we replicate the algorithm simulating the nonresponse mechanism for *π*_*z*_ = 0.2 (*π*_*z*_ remains constant across the phases), *π*_*m*_(1) = 0.85, and *π*_*m*_(2) = 0(0.1)0.9. First we run 1000 simulations and plot the bias index $r_{\text{HH}}=E({\bar{y}}_{\text{HH}})/\bar{y}$. As expected, for *π*_*m*_(2) = 0 we have *r*_HH_ = 1, that is, the nonresponse bias is eliminated (Fig 3). For *π*_*m*_(2) > 0 we obtain *r* < 1, which means that using the estimator ${\bar{y}}_{\text{HH}}$ leads this time to an underestimation. This underestimation exceeds 70% for *π*_*m*_(2) = 0.9 (Fig 3).

Again, for the two-phase sampling design, the nonresponse bias is not affected by the sampling fractions used. If we set *ν*_*m*_ = *ν*, with *ν* = 0.1(0.1)0.5, we obtain the same results, except that there are Monte Carlo fluctuations (see Table 1).

**Table 1:** For the simulated population, bias index of the Hansen & Hurwitz estimator as a function of the nonresponse rate at the last phase, with increasing sampling fractions.

π_*m*_(2)	ν = 0.1	ν = 0.2	ν = 0.3	ν = 0.4	ν = 0.5
0.0	1.006	1.001	1.010	0.996	0.999
0.1	0.944	0.937	0.936	0.931	0.930
0.2	0.862	0.861	0.863	0.863	0.863
0.3	0.794	0.780	0.790	0.798	0.791
0.4	0.692	0.707	0.720	0.716	0.716
0.5	0.633	0.626	0.632	0.636	0.634
0.6	0.545	0.556	0.555	0.553	0.552
0.7	0.464	0.469	0.470	0.469	0.466
0.8	0.378	0.375	0.378	0.375	0.377
0.9	0.288	0.279	0.285	0.279	0.280

Bias index $r_{\text{HH}}=E({\bar{y}}_{\text{HH}})/\bar{y}$ based on 1000 simulations of the nonresponse mechanism, for *π*_*z*_ = 0.2, *π*_*m*_(1) = 0.85, and *π*_*m*_(2) = 0(0.1)0.9. The sampling fraction varies as *ν* = 0.1(0.1)0.5, with *ν*_*m*_ = *ν*. Details in the text.

**Fig 4:**
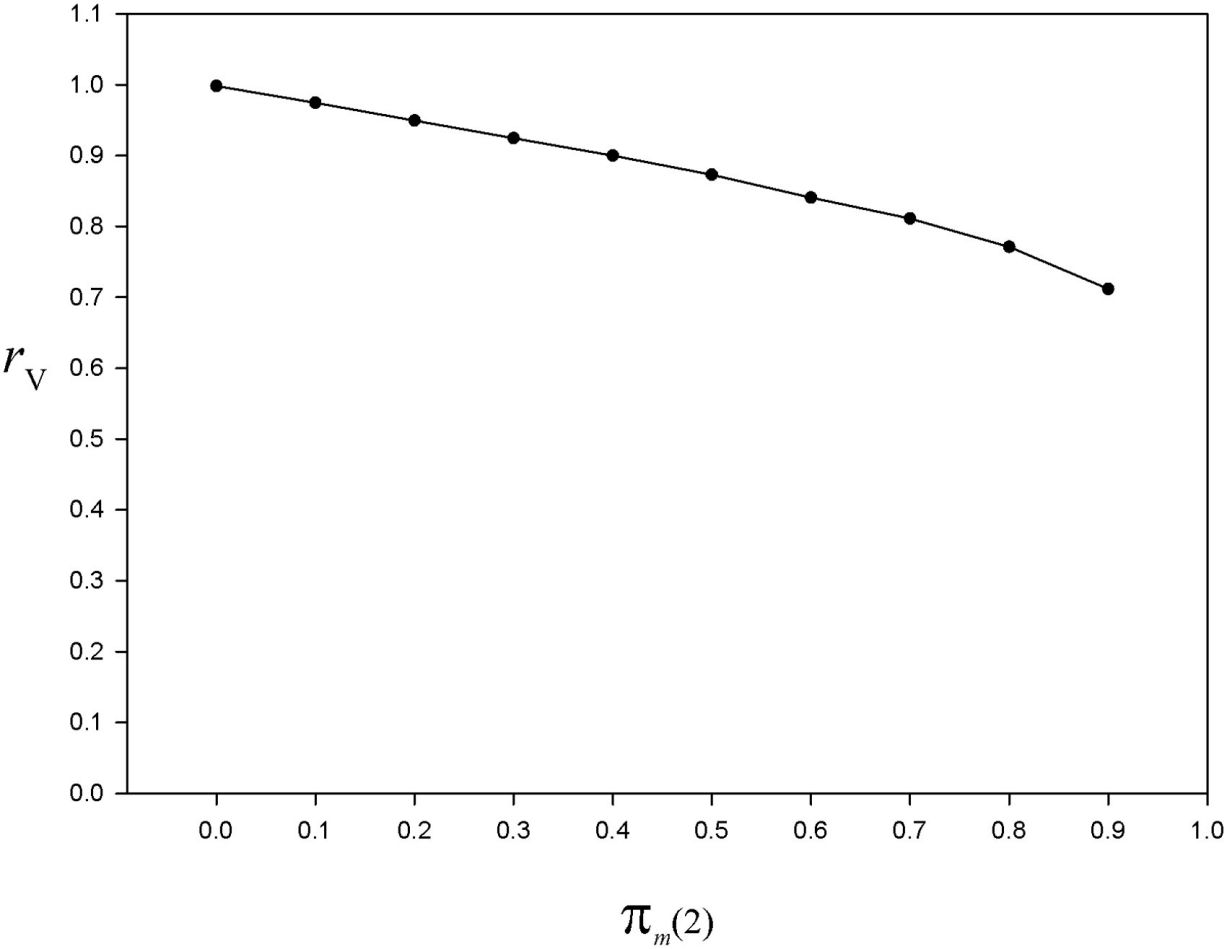
For the simulated population, bias index of the sampling variance estimator as a function of the nonresponse rate at the last phase. Curve of the bias index $r_{\text{V}}=E\left({\hat{V}}_{p}\left({\bar{y}}_{\text{HH}}\right)\right)/V_{\text{MC}}\left({\bar{y}}_{\text{HH}}\right)$ based on 1 000 000 simulations of the nonresponse mechanism, for *π*_*z*_ = 0.2, *π*_*m*_(1) = 0.85, and *π*_*m*_(2) = 0(0.1)0.9. Details in the text.

Besides the behavior of the estimator ${\bar{y}}_{\text{HH}}$, we are also interested in that of the sampling variance estimator, that is ${\hat{V}}_{p}\left({\bar{y}}_{\text{HH}}\right)$ (12). Thus, in a second time, we run 1 000 000 simulations and plot the bias index $r_{\text{V}}=E\left({\hat{V}}_{p}\left({\bar{y}}_{\text{HH}}\right)\right)/V_{\text{MC}}\left({\bar{y}}_{\text{HH}}\right)$ where $V_{\text{MC}}\left({\bar{y}}_{\text{HH}}\right)$ is a Monte Carlo approximate of $V_{p}\left({\bar{y}}_{\text{HH}}\right)$. As expected, we have *r*_V_ = 1 for *π*_*m*_(2) = 0 (the sampling variance estimator is unbiased when nonresponse rate at the second phase is null) (Fig 4). When *π*_*m*_(2) > 0, then we obtain *r*_V_ < 1, that is, using the estimator ${\hat{V}}_{p}\left({\bar{y}}_{\text{HH}}\right)$ leads to an underestimation of the sampling variance.

### 5.4 Nonresponse bias attenuation under multiphase design

**Fig 5:**
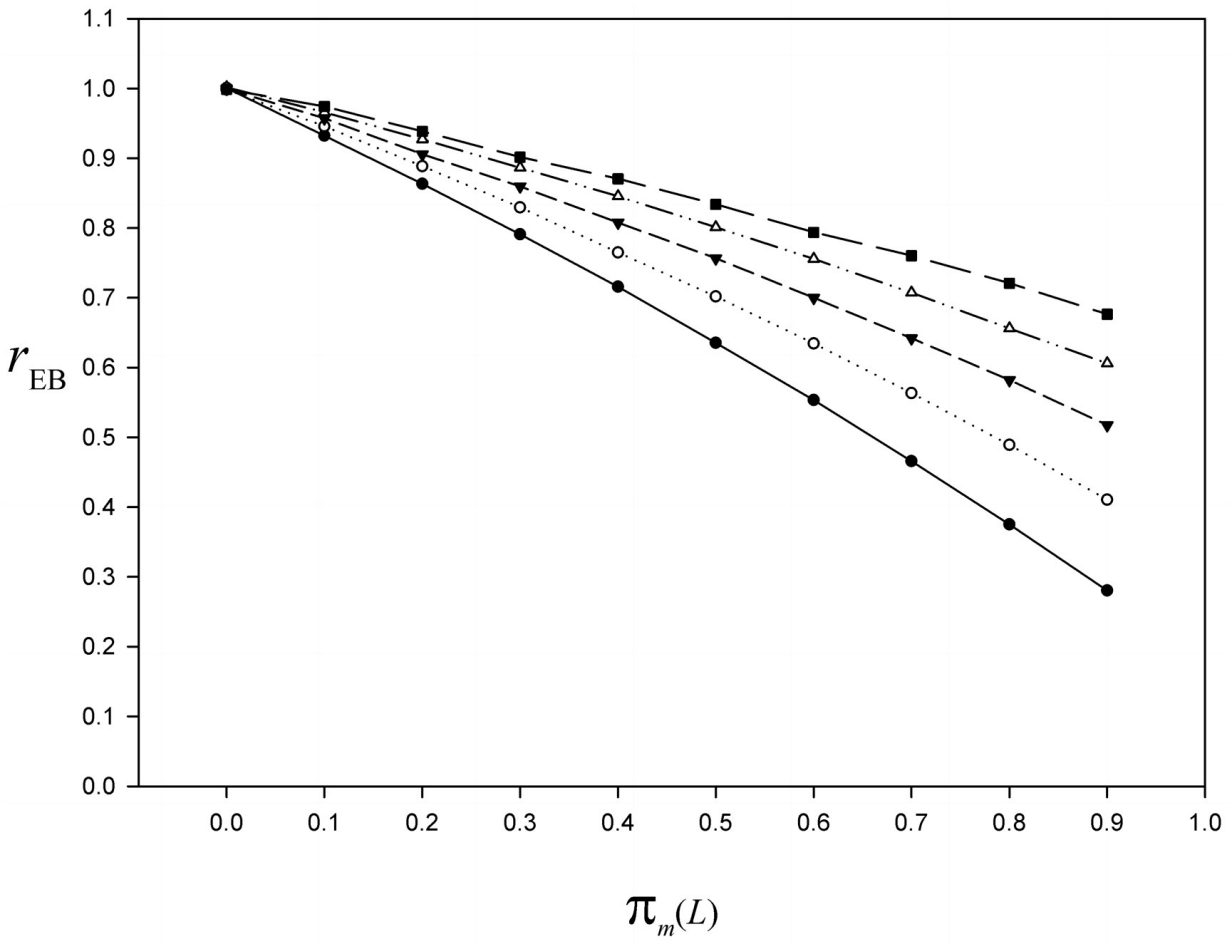
For the simulated population, bias index of the El-Badry estimator as a function of the nonresponse rate at the last phase. Curve of the bias index $r_{\text{EB}}=E({\bar{y}}_{\text{EB}})/\bar{y}$ based on 10 000 simulations of the nonresponse mechanism, for *L* = 2(1)6, *π*_*z*_ = 0.2, *π*_*m*_(*i*) = 0.85 for 1 ≤ *i* < *L*, and *π*_*m*_(*L*) = 0(0.1)0.9 (*black dots* *L* = 2; *white circles* *L* = 3; *black triangles down* *L* = 4; *white triangles up* *L* = 5; *black squares* *L* = 6). Details in the text.

In this section, we first examine the effect of the number of phases *L* (section 3) on the nonresponse bias attenuation. Let *π*_*m*_(*i*) denote the values of *π*_*m*_ at the *i*-th phase (1 ≤ *i* ≤ *L*). For the same finite population as in section 5.2, under a *L*-phase sampling design with *L* = 2(1)6, and sampling fractions *ν*_*i*_ = 0.5 (1 ≤ *i* ≤ *L*), we replicate the algorithm simulating the nonresponse mechanism for *π*_*z*_ = 0.2 (*π*_*z*_ remains constant across the phases), *π*_*m*_(*i*) = 0.85 for 1 ≤ *i* < *L*, and *π*_*m*_(*L*) = 0(0.1)0.9. We run 10 000 simulations and plot the bias index $r_{\text{EB}}=E({\bar{y}}_{\text{EB}})/\bar{y}$. As expected, whatever the number of phases *L*, for *π*_*m*_(*L*) = 0 we have *r*_EB_ = 1, that is, the nonresponse bias is eliminated (Fig 5). As previously, for *π*_*m*_(*L*) > 0 we obtain *r*_EB_ < 1, which means that the estimator ${\bar{y}}_{\text{EB}}$ is biased downwards. The underestimation decreases as the number of phases *L* increases (Fig 5).

**Fig 6:**
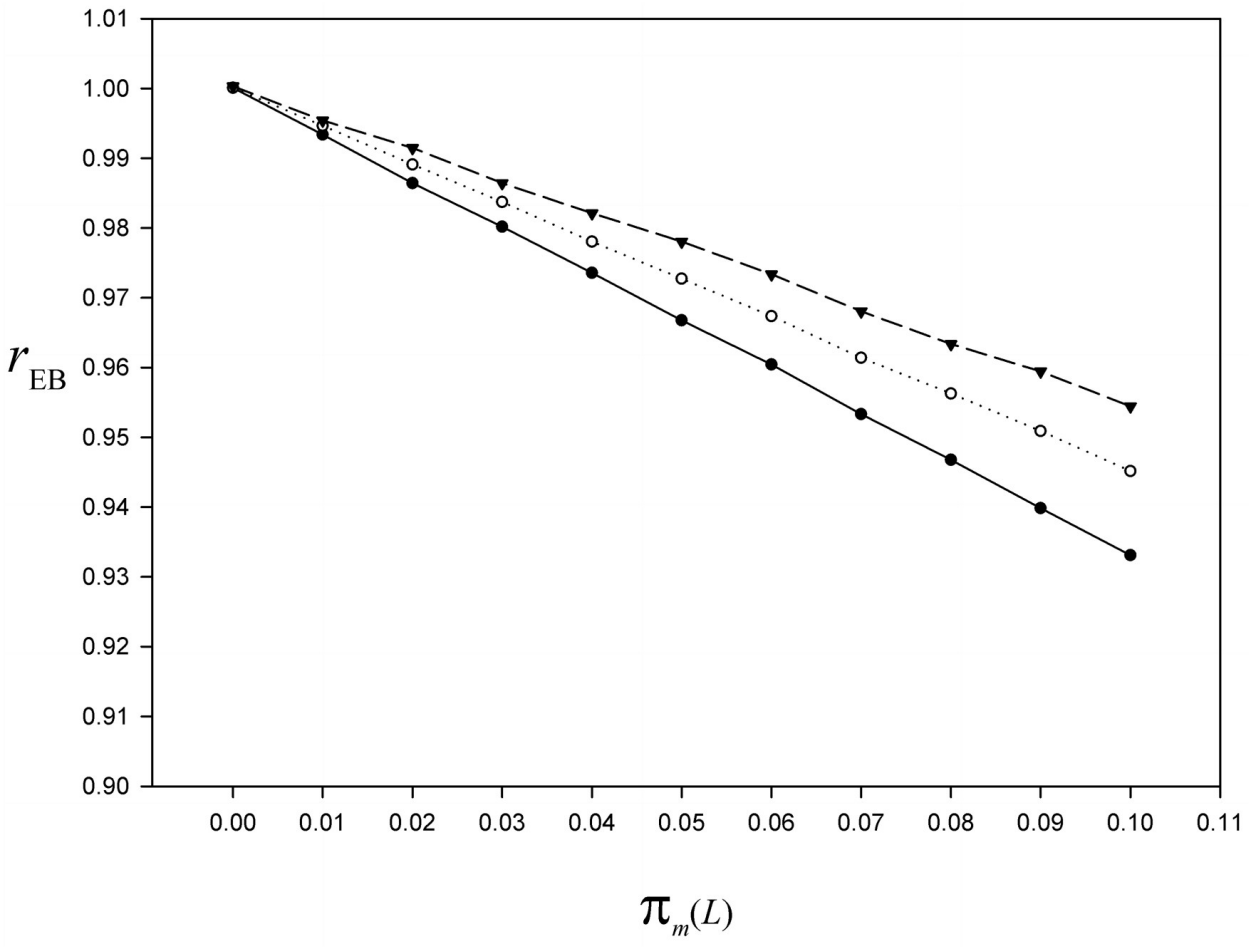
For the simulated population, bias index of the El-Badry estimator as a function of the nonresponse rate at the last phase (detail for moderate nonresponse rates). Curve of the bias index $r_{\text{EB}}=E({\bar{y}}_{\text{EB}})/\bar{y}$ based on 100 000 simulations of the nonresponse mechanism, for *L* = 2, 3, 4, *π*_*z*_ = 0.2, *π*_*m*_(*i*) = 0.85 for 1 ≤ *i* < *L*, and *π*_*m*_(*L*) = 0(0.01)0.1 (*black dots* *L* = 2; *white circles* *L* = 3; *black triangles down* *L* = 4). Details in the text.

We now examine the underestimation for moderate values of *π*_*m*_(*L*), for instance up to 0.1. See Fig 6 for the plot of *r*_EB_ after 100 000 simulations, for *L* = 2, 3, 4.

**Fig 7:**
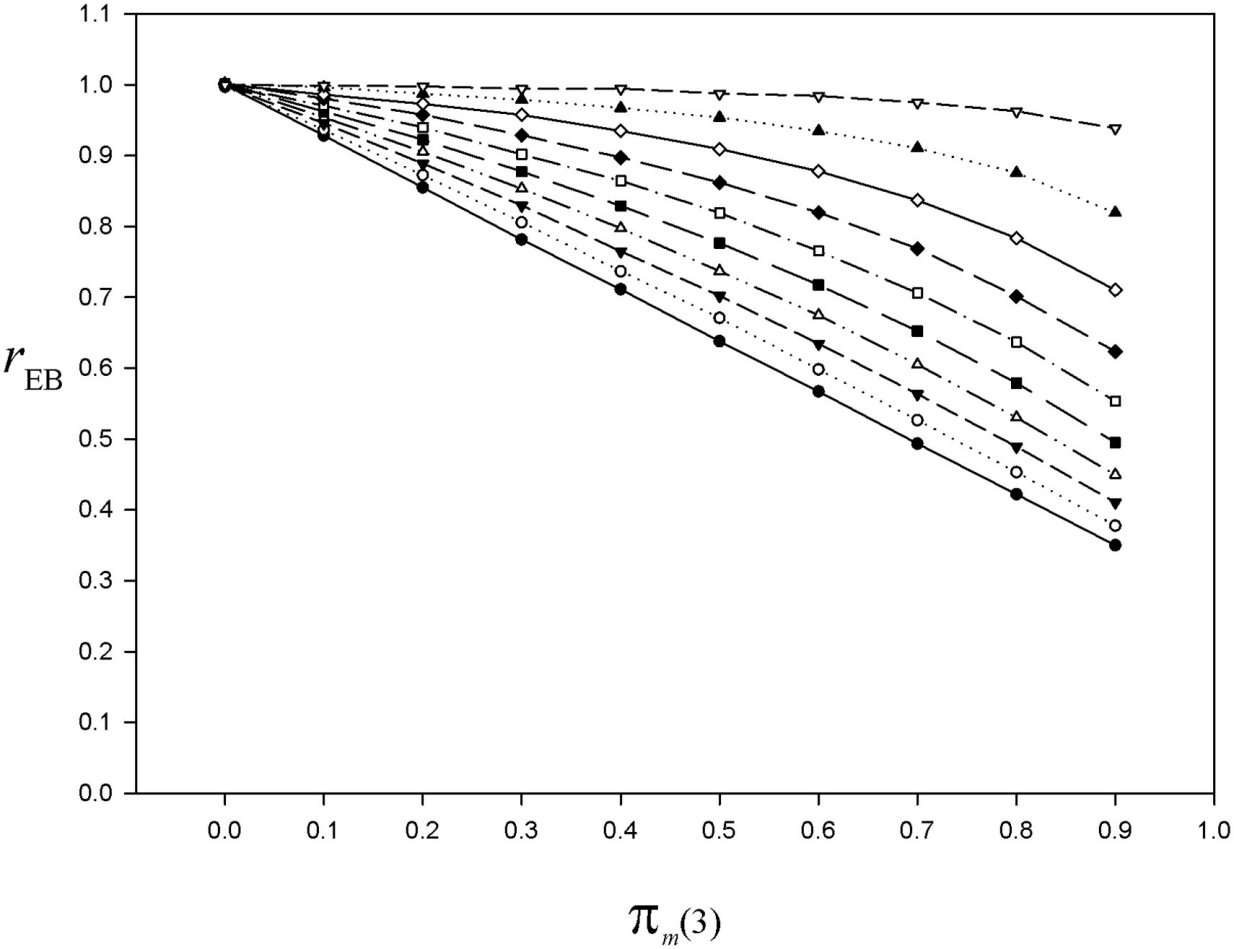
For the simulated population, bias index of the El-Badry estimator as a function of the nonresponse rate at the last phase, with varying values of π_*z*_. Curve of the bias index $r_{\text{EB}}=E({\bar{y}}_{\text{EB}})/\bar{y}$ based on 10 000 simulations of the nonresponse mechanism, for *L* = 3, *π*_*m*_(*i*) = 0.85 for 1 ≤ *i* < *L*, *π*_*m*_(*L*) = 0(0.1)0.9, and *π*_*z*_ = 0(0.1)0.9 (*black dots* *π*_*z*_ = 0; *white circles* *π*_*z*_ = 0.1; *black triangles down* *π*_*z*_ = 0.2, *white triangles up* *π*_*z*_ = 0.3, *black squares* *π*_*z*_ = 0.4, *white squares* *π*_*z*_ = 0.5, *black diamonds* *π*_*z*_ = 0.6, *white diamonds* *π*_*z*_ = 0.7, *black triangles up* *π*_*z*_ = 0.8, *white triangles down* *π*_*z*_ = 0.9). Details in the text.

Second, taking for example the case *L* = 3, we illustrate the variation of the underestimation according to the value of *π*_*z*_. We replicate the algorithm simulating the nonresponse mechanism as previously except that, although *π*_*z*_ continues to remain constant across the phases, now it varies as *π*_*z*_ = 0(0.1)0.9. For each value of *π*_*z*_, we run 10 000 simulations and plot the bias index $r_{\text{EB}}=E({\bar{y}}_{\text{EB}})/\bar{y}$. The underestimation is maximum for *π*_*z*_ = 0 and decreases as *π*_*z*_ increases since it is offset by the potential upward nonresponse bias (which increases in magnitude with *π*_*z*_) (Fig 7). Thus, in the hypothetical case where *π*_*z*_ would be very high, the nonresponse bias would be strongly attenuated even with a low response rate at the last phase.

## Discussion

In surveys, choices concerning the collecting mode (mail, web, phone), length, content, organization, wording and color of the questionnaire, type of outgoing postage, type of return postage, content of cover letter, the way the survey is publicized to surveyed people, the stakeholders in charge of the survey, are all factors susceptible of having an impact on final response rate and, hence, on potential nonresponse bias. Regarding questionnaire design, the simpler it is the most responses can be expected. There is however a limit to simplification and even a questionnaire which appears simple to the staff in charge of a hunting bag survey may be misunderstood by some of the surveyed people, and therefore may lead to nonresponse. In parallel, we live in a society experiencing an increasing demand for information. Consequently, more and more people are asked to participate in surveys, and they may increasingly see this as a burden. Therefore, people may become less and less inclined to cooperate [32]. This phenomena also holds for hunters of course. Since nonresponse is a psychosociological phenomena, it is very difficult to forecast which option or combination of options will have a significant positive impact on response rate. In practice, minimizing the anticipated nonresponse when designing the questionnaire requires a series of trial and error tests. For the very important issue of questionnaire design and administration, the reader is referred to [14, 72–75].

Skalski & Millspaugh [76] state that “Estimating game harvest is among the most important activities of wildlife management agencies”. Even though web-based systems tend to develop, usually hunting bag surveys still rely in part or totality upon self-administered mailed questionnaires. Despite the prominence of the nonresponse bias issue in mail hunting bag surveys [10, 14, 30, 41, 77–79], few papers have been published about statistical remedies facing this problem in this specific context, and fewer still wildlife agencies have taken this problem into account seriously and adequately. Some reports (e.g. [8]) or proceedings (e.g. [37]) on this topic do exist, but seem to have fallen into oblivion or are difficult to access. Accordingly, this paper is an opportunity to bring back to light the issue and to provide a practical, statistically sound solution, namely subsampling among nonrespondents in the framework of multiphase sampling for stratification.

In this paper we recalled the strategy proposed by Hansen & Hurwitz [48] and its generalization to any number of phases carried out by El-Badry [64]. At least in North America, the unbiased estimator introduced by Hansen & Hurwitz [48] is known (see [77], Footnote 3, [14, 80], [81], p. 239) and actually used (see [37] and [82]). Unfortunately, an unbiased sampling variance estimator was not necessarily used. For instance, after correcting the misformulated sampling variance printed in [44] (see our remark in section 2.3), Taylor et al. [82] simply used it by substituting sample estimates to population parameter values, which by no means leads to an unbiased estimator. Oddly enough, no sampling variance estimator is given in a number of books dealing with the Hansen-Hurwitz’s method (see for instance [53, 54, 65, 68, 83, 84]).

The generalization by El-Badry [64] was cited by Filion [14] but, to our knowledge, his strategy has not been used in the context of hunting bag surveys until the last French nationwide hunting bag survey [85, 86]. MacDonald & Dillman [10] referred to El-Badry [64] but only for introduction generalities about nonresponse. Again, unfortunately for the practitioner, a sampling variance estimator was not given by El-Badry [64], nor in any of the rare books, theses or articles which address this design beyond the mere mention of its existence (see [87, 88], [62], pp. 104-105, [83], pp. 511-512, [65], p. 122, [67], p. 61 and p. 66, [68], pp. 406-409). We have filled this gap by providing an unbiased sampling variance estimator for any number of phases (section 3.4). We also provided the detailed expression of the sampling variance estimators for two- and three-phase sampling (see S1 Appendix), since such numbers of phases are the most likely to be used in practice, based on economic and logistical considerations, but it is safer to implement in a programming language the general expression we gave. We hypothesize that the lack of sampling variance estimator may have contributed to El-Badry’s sampling strategy not becoming a regular element of wildlife agencies’ toolbox.

For unbiased estimation of the total (or mean) and sampling variance, the El-Badry’s sampling strategy requires a 100% response rate at the last phase of the multiphase sampling design, that is, when the hunters of a subsample drawn from the last mailing wave nonrespondents are interviewed (usually by phone). However, in practice, whatever the number of mailing waves, at the last phase the response rate cannot be 100%. Accordingly, the nonresponse bias cannot be totally eliminated by the multiphase sampling design. Nevertheless, a certain amount of bias attenuation should result from using the total estimator under the El-Badry’s sampling strategy, depending on the nonresponse rate at the last phase *L* and potential magnitude of the nonresponse bias. To document this topic of paramount practical importance, we relied on Monte Carlo simulations. We found that a negative bias is induced by the nonresponse occurring at the last phase, both in estimating the mean (or total) (Figs 3 and 5) and the sampling variance (Fig 4). Moreover, the Monte Carlo study showed that the nonresponse bias attenuation (that is, when *π*_*m*_(*L*) is not 0) increases jointly with the number of phases (Fig 5). Actually, increasing the sampling effort with the aim of attenuating the nonresponse bias is only possible by increasing the number of phases. The fact that increasing the sampling size at any phase has no effect on the nonresponse bias should be recalled here since some authors saw this as a way to reduce the nonresponse bias (e.g. [89], p. 30).

Our Monte Carlo simulations also illustrate the fact that, in case of a very large potential nonresponse bias (caused by the conjunction of high values both of *π*_*m*_ and *π*_*z*_), the El-Badry’s sampling strategy leads to an important bias attenuation, even though the response rate at the last phase is not very high (Fig 7). As we assume in practice a moderate value for *π*_*z*_, from the Monte Carlo case study we advocate that the response rate at the last phase should not be lower than 90%. Whatever the number of phases (i.e. especially with *L* = 2), a moderate underestimation of the nonresponse bias (say a maximum of 5%) is only achieved with a response rate of at least 93% in the last phase (Fig 6).

Although the cost/precision balance is an important topic, it would carry too far in this article to address these issues (see [48, 50, 62, 64, 69, 88], [52], pp. 371-372, and [53], pp. 977-979). However, two- or three-phase sampling are generally acceptable on economic and logistical grounds, and seems to provide a reasonable trade-off between additional mailing costs for multiple mailing waves, bias attenuation and increase of the sampling variance (since the sampling variance increases with the number of phases). In the case of a postseason survey conducted with an annual periodicity, in our experience, the success of the strategy we advocate in this article depends on several critical factors. First, the quality of the sampling frame is one of the most relevant prerequisites, both in terms of coverage and correctness of contact information (postal address and phone numbers). Second, it is of the utmost importance that designing the questionnaire (either paper- or web-based) as well as receiving and processing the questionnaires be accomplished by the wildlife agency itself. We strongly advise against relying on unskilled organizations regarding hunting surveys such as market research organizations or opinion poll organizations: only printing and mailing could be outsourced. Third, the timing of mailing waves and of the last phase phone interview must be carefully planned and respected. Fourth, entry and control of the hunting bags reported (questionnaires completed) must be carried out on a continuous flow basis. Of course, is it possible that one or two surveys be necessary before entering in a perfectly mastered routine, but we think that the quality of the hunting bag estimating scheme worths these efforts.

The scope of the method reintroduced in this paper is broader than that of hunting bags surveys, and naturally covers other surveys that also have nonresponse issues [90]. By contrast, in terms of nonresponse bias, the recommendations must be domain-specific and cannot be automatically applied to other fields. In the present article, the Monte Carlo simulations were based on a nonresponse mechanism that makes sense in the field of hunting bag surveys, but which may have no phenomenological validity in another domain. The nonresponse mechanism we proposed (section 4.1) is simple but realistic enough to be useful. In this mechanism, we retain two parameters related to the nonresponse bias: the propensity to not respond (*π*_*m*_) and, among the nonrespondents, the propensity to not respond because of a null harvest (*π*_*z*_). In this situation, the nonresponse bias is equivalent to an undercoverage bias of the stratum *U*_0_ (null hunting bags) by the set of respondents (see Fig 1).

It is conceivable that another nonignorable cause of nonresponse would be the fact for a hunter to have a very high hunting bag (which he/she would not be ready to disclose). Nevertheless, we advocate that this cause can be neglected compared to the issue of null bags, for at least two reasons. First, it seems unlikely that most of the very successful hunters do not respond because of their success. Indeed, it would be in contradiction with the fact that hunters tend overstating their bags for prestige or pride reasons, even though the survey was announced as anonymous (it is likely that some of the respondents do not believe this anonymity claim, because they received a nominative mail). Hunters do not seem to hesitate to report very high hunting bags, even when they exceed existing legal limits. Second, the overwhelming majority of hunters have a null harvest for a given game species, either because they were inactive or unsuccessful. For instance, in France, even for the most harvested wild bird species (i.e. without released birds), namely the common wood pigeon (*Columbia palumbus*), the proportion of hunters with null harvest was estimated at 78% according to the last survey (2013-2014 hunting season). Moreover, the proportion of hunters with null harvest for all allowed game species (about 90 species in France) was estimated at 30%. By contrast, very successful hunters are rarer.

Regarding our simulations, note that we found several other algorithms implementing the nonresponse mechanism described in section 4.1, all equivalent with reference to the nonresponse bias. We retained the algorithm that fit exactly the context of the Monte Carlo study under consideration, namely the framework of multiphase sampling for stratification. For other studies related to the same nonresponse mechanism, one of the other algorithms could be used, and will be documented at this occasion.

Although we cannot claim the generality of the finite population we used as an example for the Monte Carlo study, it is nevertheless rooted in reality. Indeed, we have set the population size to 10 000, which is the order of magnitude of the average number of (potentially) active hunters in a French department (a department is a mid-scale administrative entity used as a geographical stratum in the last nationwide French hunting bag survey, see [85, 86]). The two parameters specifying the superpopulation model *ξ* are approximately the values for Eurasian teal (*Anas crecca*) hunting bags, estimated from the last nationwide French hunting bag survey (i.e. national-scale estimates). The value *π*_*m*_ = 0.85 corresponds approximately to the observed mean nonresponse rates (i.e. among geographical strata) for each of the two mailing waves in the last nationwide French hunting bag survey (average nonresponse rate of 86% for the first mailing wave, and of 88% for the second, [85]). Lastly, the value *π*_*z*_ = 0.2 corresponds to the order of magnitude of the proportion for nonrespondents in the second mailing wave, who declared by phone in the (last) third phase that they did not respond previously because of a low or null harvest (17%, see [91], encadré, p. 6). By using this example—which leads to an overestimation of about 20% with the usual estimator (see Fig 2)—we have been realistic in that overestimates of about 20% (or more) seem not to be exceptional [23, 41].

If we consider for instance a three-phase sampling design, and a response rate at the last phase greater than 90%, we can expect an underestimation of about 5% or less (Fig 6). In such a case, in terms of cost/benefit ratio, it is useless resorting to this sampling strategy when uni-phase SRSWOR leads to an overestimation equal or less than 5% (for the range of *π*_*m*_ and *π*_*z*_ values, see the contour level 1.05 on Fig 2). If we set *π*_*z*_ = 0.20, it might not be very useful to use this sampling strategy for instance when *π*_*m*_ = 0.40, since the uni-phase SRSWOR leads to an overestimation of about 9% (Fig 6). This might be the case for the Finnish hunting bag survey for which the response rate is currently about 60% (Leena Forsman, pers. comm.). At this stage of the reflexion, the genuine question is whether a given overestimation magnitude is inconsequential or not for the purpose at hand. As Chapman et al. [8] wrote, “The definition of any given error as ‘inconsequential’ is also quite relative, as under a different set of circumstances or in a different application such an error magnitude might not be inconsequential at all”. In accordance with Fig 2, we agree with the recommendation for a 85% response rate to minimize the impact of nonresponse [79]. It must however be acknowledged that such a high response rate currently seems to be the exception rather than the rule. For instance, it is conversely the nonresponse rate that reached 85% at each mailing wave in the last French nationwide hunting bag survey. In this circumstance, the three-phase sampling design proved to be essential for attenuating a nonresponse bias that would otherwise have been far from negligible.

In the field of wildlife management, even in the most advanced countries (e.g. the U.S. or Canada, but see [1–3] for a discussion of this assertion), the nonresponse bias issue is not always addressed by the producers of hunting bag statistics [92]. In principle, one may suggest different reasons for this, which are non exclusive from each other: (i) poor awareness of the problem, (ii) financial or time constraints, (iii) hunting statistics as the result of a pure administrative request rather than a scientific question. A fourth reason could be that the variable of interest often is the trend in hunting bags rather than absolute hunting bag size (e.g. [93]), which may make more sense given the multiple biases potentially affecting absolute bag estimates [24]. As written by Wright [13]: “The important problem then is to estimate changes in the types and magnitude of the biases between years”. Some authors claim that there is evidence that the nonresponse bias changes between years (e.g. [41]). At this stage, we have no general certainty, and careful case-by-case studies are needed. For trend assessment, in practice we only need that the nonresponse bias can be held relatively constant in time. It is especially of utmost importance that the nonresponse bias does not itself show a trend (up or down) over time, otherwise it will be impossible to interpret the presence/absence of a trend as representative of that of the actual hunting bags.

For trend assessment, under the nonresponse mechanism we proposed in the context of hunting bag surveys, if *π*_*m*_ may be considered as approximately constant, this condition must also hold for *π*_*z*_ since these two probabilities play a symmetrical role in producing nonresponse bias. It is well known that the response rate shows a general decreasing trend over time, whatever the topic of the survey at hand (see for instance [47], Section 2.2). Such a situation also holds for hunting bag surveys. For instance, for the Illinois waterfowl hunter state survey, the overall response rate was 70-83% for the years 1982-1992 [94] and decreased to 44% for the 2015-2016 season [95]. In Finland, the response rate to the nationwide hunting survey was about 75% in 2012 and is currently about 60% (Leena Forsman, pers. comm.). However, it is likely that there is a threshold below which the response rate cannot decline further, depending on several factors such as the geographical scale of the survey, its periodicity, advertising for the survey and so on. Thus, at least in certain circumstances, we can think that the nonresponse rate may remain stable (the response rate cannot fall indefinitely to zero) and thus it seems possible to consider *π*_*m*_ as approximately constant (but only from a certain point in time that we do not know in advance). There remains the issue of *π*_*z*_. Although changes in the hunting conditions may lead to a change in the proportion of null harvest among hunters taking a licence (i.e. potentially active hunters), this does not necessarily imply a change in *π*_*z*_, for the propensity to report null harvest may be under the influence of several psychosociological processes. Anyway, the approximately constant nonresponse bias in time is a key assumption we think wiser not to make. Instead, it is safer using the adequate El-Badry’s sampling strategy, ensuring a very moderate nonresponse bias in the estimation.

From a sampling point of view, hunting bag estimates can be established on the basis of a sample survey or *a census survey* (or simply, *a census*). A census may be viewed as a the limiting case of a sample survey, that is, when all the members of the frame are surveyed (a complete enumeration of all potentially active hunters). Whatever the type of survey, responding to a hunting bag questionnaire may be mandatory or on a voluntary basis only. When reporting hunting bags is mandatory, a fine may be provided for by the legislation in case of non-reporting (e.g. [96] for Denmark). A more effective incentive is obtained by conditioning the delivery of the license for a new hunting season on reporting the hunting bag for the previous hunting season. For instance, with such a measure, Denmark nowadays reaches a hunting bag return rate of almost 100% [21]. In practice, conditioning hunting on response is usually restricted to census. In case of a census, even when the reporting of hunting bags is mandatory, generally in the absence of a fine or prosecution such as mentioned above, the response rate is not 100%, according to hunters’ compliance, which varies both at the individual and cultural level, at the nationwide or regional scale. A high response rate needs both strong adherence to the rule by the active hunter population and effective law enforcement by the authorities. So even though in some countries the response rate is nowadays close to 100% (e.g. Denmark, Norway), for a number of countries where hunting bag reporting is mandatory, the nonresponse bias issue is still relevant. Hence, in the case of a census with a moderate response rate, the El-Badry’s sampling strategy might be applied (just consider the first phase sampling fraction *ν*_1_ = 1).

From a logistic point of view, using two or more mailing waves in hunting surveys was common in North America some time ago [7, 10, 14, 22, 26, 38, 43, 77, 79, 80, 94]. However, efforts were not always made to differentiate between the responses of the different waves [29], and the followup was only dedicated to gather more responses. For instance, Anderson et al. [94] report that in the case of the Illinois Waterfowl Hunter Survey for the years 1982-1992, the initial mailing and 2 followups to nonrespondents generated response rates of 70-83%. Under such conditions, the total hunting bag is often estimated by pooling the responses of the successive mailing waves, and possibly those gathered by telephone follow-up (e.g. [26]). When the responses from successive mailings are tabulated separately, some authors rely on the assumption that there exists a continuum of respondent types which range from highly motivated to unmotivated individuals, that is to say, a linear increase in nonresponse bias with successive waves. Under this assumption, they fit a regression model aimed at correcting for nonresponse bias [10, 77], following an idea that goes back at least to Clausen & Ford [40]. We are not convinced by this approach. First, in accordance with Atwood [7], in mailing follow-up we think that “differences exhibited in data from successive waves of requests must be attributed to errors other than nonresponse errors”. Besides, Sen [97] suggested that “the average kill per hunter may not change appreciably when successive reminders are used to reduce nonresponse”. Second, the usefulness of a regression model fit on the basis of very few points can be questioned. Anyway, in this context, using a regression model deserves a thorough study, which could be the aim of another paper. Finally, at this stage of our knowledge, again, we advocate the El-Badry’s sampling strategy especially because no assumptions are required. Moreover, a design-based approach allows to manage a great number of game species in the same survey, without having to assume that the nonresponse bias affects them all the same way—which is certainly not the case [37]—and without having to treat each species separately, in the sense that the same estimators apply to all in an automatic way.

If one considers as relevant the nonresponse mechanism we proposed in this paper, then there is a need to document the propensity of non respondents to not respond because of a null harvest (*π*_*z*_), in addition to the propensity to not respond (*π*_*m*_). Communication and educational actions towards hunters are needed for decreasing both parameters. This must be done through different channels, preferably at a local scale, by hunter’s clubs and organizations. Anyway, we advocate that the El-Badry’s sampling strategy is a good way to tackle the nonresponse bias issue, provided that the nonresponse rate at the last phase remains low. Needless to say, this sampling strategy has no effect on the other nonsampling biases such as the misclassification bias mentioned in the introduction. The negligible influence of misclassification error on the final estimates cannot be taken for granted, although some studies seem to be reassuring about identification errors (e.g. [98]). On the contrary, for some game species, it can be an important source of bias which deserves more studies (see [21] for a recent contribution). Some room for improvement hence remains to ensure the quality of hunting bag surveys but, in total accordance with Pendleton [78], we think that prerequisites are relying on a sampling frame of high quality (good coverage, accurate postal addresses and phone numbers) and attenuating the nonresponse bias using repeated sampling of nonrespondents.

Adaptive harvest management is gradually becoming the norm in wildlife agencies, following the very successful example of North Americans for waterbird hunting [99–101]. When hunting bag estimates are inputs in adaptive harvest management models, attenuating the nonresponse bias becomes of overwhelming importance, otherwise overestimates will be taken into account in the calculations, with the risk of misleading conclusions and unsuitable management recommendations. A recent analysis suggests adaptive harvest management is achievable even with minimum data availability, but regular and robust estimates of hunting bags are among those few absolute prerequisites [6]. Subsampling the nonrespondents in the framework of multiphase sampling for stratification is a usable solution, offering a good protection against high nonresponse bias.

## Supporting information

S1 Appendix Statistical and formal complements. (PDF)
