# Supplementary material for: Attenuating the nonresponse bias in hunting bag surveys: The multiphase sampling strategy
Source: PLoS One. 2019 Mar 15;14(3):e0213670. doi: 10.1371/journal.pone.0213670 (PMC6420046; doi:10.1371/journal.pone.0213670)
Supplement: S1 Appendix — (PDF) [file pone.0213670.s001.pdf]

Statistical and formal complements

A.1 Sampling variance estimator for $L = 2$

For two-phase sampling, the design can be summarized by the scheme:

$$\begin{array}{ccccccc} U & \xrightarrow{\text{SRSWOR}} & s_1 & \rightarrow & r_1 & & \\ & & & \searrow & & & \\ & & & & m_1 & \xrightarrow{\text{SRSWOR}} & r_2 \end{array} \tag{1}$$

From our general expression for the sampling variance estimator, with $L = 2$ we obtain:

$$\begin{aligned} \widehat{\mathrm{V}}_p\left(\hat{t}_{\mathrm{EB}}\right) = & \frac{N(N-n_{s_1})}{n_{s_1}(n_{s_1}-1)}\left(z_1 - \frac{1}{n_{s_1}}t_1^2\right) \\ & + \frac{N(N-1)}{n_{s_1}(n_{s_1}-1)}\left[n_{m_1}^2\left(\frac{1}{n_{s_2}} - \frac{1}{n_{m_1}}\right)S_{r_2}^2\right] \end{aligned} \tag{2}$$

with

$$t_2 = \sum_{k \in r_2} y_k \tag{3}$$

$$t_1 = \sum_{k \in r_1} y_k + \frac{n_{m_1}}{n_{s_2}} t_2 \tag{4}$$

and

$$z_2 = \sum_{k \in r_2} y_k^2 \tag{5}$$

$$z_1 = \sum_{k \in r_1} y_k^2 + \frac{n_{m_1}}{n_{s_2}} z_2 \tag{6}$$

A.2 Sampling variance estimator for $L = 3$

For three-phase sampling, the design can be summarized by the scheme:

$$\begin{array}{ccccccccc} U & \xrightarrow{\text{SRSWOR}} & s_1 & \rightarrow & r_1 & & & & \\ & & & \searrow & & & & & \\ & & & & m_1 & \xrightarrow{\text{SRSWOR}} & s_2 & \rightarrow & r_2 \\ & & & & & & & \searrow & \\ & & & & & & & & m_2 \xrightarrow{\text{SRSWOR}} r_3 \end{array} \tag{7}$$

From our general expression for the sampling variance estimator, with $L = 3$ we obtain:

$$\begin{aligned} \widehat{\mathrm{V}}_p\left(\hat{t}_{\mathrm{EB}}\right) = & \frac{N(N-n_{s_1})}{n_{s_1}(n_{s_1}-1)}\left(z_1 - \frac{1}{n_{s_1}}t_1^2\right) \\ & + \frac{N(N-1)}{n_{s_1}(n_{s_1}-1)}\left\{\frac{n_{m_1}(n_{m_1}-n_{s_2})}{n_{s_2}(n_{s_2}-1)}\left(z_2 - \frac{1}{n_{s_2}}t_2^2\right)\right. \\ & \left. + \frac{n_{m_1}(n_{m_1}-1)}{n_{s_2}(n_{s_2}-1)}\left[n_{m_2}^2\left(\frac{1}{n_{s_3}} - \frac{1}{n_{m_2}}\right)S_{r_3}^2\right]\right\} \end{aligned} \tag{8}$$

with

$$t_3 = \sum_{k \in r_3} y_k \tag{9}$$

$$t_2 = \sum_{k \in r_2} y_k + \frac{n_{m_2}}{n_{s_3}} t_3 \tag{10}$$

$$t_1 = \sum_{k \in r_1} y_k + \frac{n_{m_1}}{n_{s_2}} t_2 \tag{11}$$

and

$$z_3 = \sum_{k \in r_3} y_k^2 \tag{12}$$

$$z_2 = \sum_{k \in r_2} y_k + \frac{n_{m_2}}{n_{s_3}} z_3 \tag{13}$$

$$z_1 = \sum_{k \in r_1} y_k + \frac{n_{m_1}}{n_{s_2}} z_2 \tag{14}$$

A.3 Distribution of n_m and n_z

Conditionally to N_M, the set-size n_m follows a hypergeometric distribution $\mathrm{P_H}(n_m|n_s, N_M, N)$. When N_M follows a two-point distribution $\mathrm{P_T}(N_M|N, \pi_m)$, then the unconditional distribution of n_m is:

$$\mathrm{P_H}(n_m|n_s, N_M, N) \bigwedge_{N_M} \mathrm{P_T}(N_M|N, \pi_m) \tag{15}$$

The pmf of this distribution can be written as:

$$\mathrm{P_{HT}}(n_m|n_s, N, \pi_m) = (1-\Theta)\cdot \mathrm{P_H}(n_m|n_s, \lfloor \mathrm{B} \rfloor, N) + \Theta \cdot \mathrm{P_H}(n_m|n_s, \lceil \mathrm{B} \rceil, N) \tag{16}$$

with $\mathrm{B} = N\pi_m$, $\Theta = \mathrm{B} - \lfloor \mathrm{B} \rfloor$, for $n_m \in \mathcal{D}_{\mathrm{HT}}(n_s, N, \pi_m)$ where:

$$\begin{aligned} \mathcal{D}_{\mathrm{HT}}(n_s, N, \pi_m) &= \mathcal{D}_{\mathrm{H}}(n_s, \lfloor \mathrm{B} \rfloor, N) \cup \mathcal{D}_{\mathrm{H}}(n_s, \lceil \mathrm{B} \rceil, N) \\ &= (\max(0, n_s + \lfloor \mathrm{B} \rfloor - N), \cdots, \min(n_s, \lceil \mathrm{B} \rceil)) \end{aligned} \tag{17}$$

Equivalently, the pmf can be written as:

$$\mathrm{P_{HT}}(n_m|n_s, N, \pi_m) = \begin{cases} \Psi \cdot \mathrm{P_H}(n_m|n_s, \lfloor \mathrm{B} \rfloor, N) & \text{for } n_m < \lceil \mathrm{B} \rceil \\ \\ \Theta \cdot \mathrm{P_H}(n_m|n_s, \lceil \mathrm{B} \rceil, N) & \text{for } n_m = \lceil \mathrm{B} \rceil \end{cases} \tag{18}$$

with:

$$\Psi = \left[1 + \Theta \left(\frac{\lceil \mathrm{B} \rceil (N - \lfloor \mathrm{B} \rfloor - n_s + n_m)}{(\lceil \mathrm{B} \rceil - n_m)(N - \lfloor \mathrm{B} \rfloor)} - 1 \right) \right] \tag{19}$$

since with our parametrization, from the relation [1] (p. 266, Equation 6.53) we have:

$$\mathrm{P_H}(x|n_s, a+1, N) = \frac{(a+1)(N-a-n_s+x)}{(a+1-x)(N-a)} \mathrm{P_H}(x|n_s, a, N) \tag{20}$$

The mean and variance are:

$$\mathrm{E_{HT}}(n_m) = n_s \pi_m = \beta \tag{21}$$

$$\mathrm{V_{HT}}(n_m) = \beta(1-\beta) + \frac{n_s(n_s-1)}{N(N-1)} \lfloor \mathrm{B} \rfloor (2\mathrm{B} - \lfloor \mathrm{B} \rfloor - 1) \tag{22}$$

In the same way, the unconditional distribution of n_z is $\mathrm{P_{HT}}(n_z|n_s, N, \pi_z\pi_m)$.

A.4 Respect of the constraint $N_Z \leq N_M$

When randomly generating N_Z such as $\mathrm{E}(N_Z) = N\pi_z\pi_m$, we must respect the constraint $N_Z \leq N_M$, which implies that the values of N, π_m and π_z must be such as to ensure $\Pr(N_Z > N_M) \simeq 0$. Using the two-point distributions $\mathrm{P_T}(N_M|N, \pi_m)$ and $\mathrm{P_T}(N_Z|N, \pi_z\pi_m)$ to generate N_M and N_Z, respectively, we examine under which conditions we have $\Pr(N_Z > N_M) > 0$.
This probability can be written:

$$\begin{aligned}\Pr(N_Z > N_M) &= (1-\Omega)\sum_{N_M=0}^{\lfloor \mathrm{A}\rfloor-1} \mathrm{P_T}(N_M|N,\pi_m) + \Omega \sum_{N_M=0}^{\lceil \mathrm{A}\rceil-1} \mathrm{P_T}(N_M|N,\pi_m) \\ &= \underbrace{\sum_{N_M=0}^{\lfloor \mathrm{A}\rfloor-1} \mathrm{P_T}(N_M|N,\pi_m)}_{\text{term 1}} + \underbrace{\Omega\cdot \mathrm{P_T}(\lfloor \mathrm{A}\rfloor|N,\pi_m)}_{\text{term 2}} \end{aligned} \tag{23}$$

where $\mathrm{A} = N\pi_z\pi_m$ and $\Omega = \mathrm{A} - \lfloor \mathrm{A}\rfloor$. To get $\mathrm{P_T}(x|N, \pi_m) > 0$, by definition we must have $x = \lfloor \mathrm{B}\rfloor$ or $x = \lfloor \mathrm{B}\rfloor + 1$ with $\mathrm{B} = N\pi_m$. As $\mathrm{A} \leq \mathrm{B}$ we have $\lfloor \mathrm{A}\rfloor - 1 < \lfloor \mathrm{B}\rfloor$ and therefore the term 1 is zero. We obtain $\mathrm{P_T}(\lfloor \mathrm{A}\rfloor|N, \pi_m) > 0$ only if $\lfloor \mathrm{A}\rfloor = \lfloor \mathrm{B}\rfloor$ (in this case we have $\Pr(N_Z > N_M) = \Omega(1-\Theta)$ with $\Theta = \mathrm{B} - \lfloor \mathrm{B}\rfloor$). Thus we get $\Pr(N_Z > N_M) > 0$ only if π_z is near 1. In practice, the respect of the constraint does not lead to a limitation of the values taken by π_z: it is sufficient to ensure that π_z be not too close to 1.

A.5 Respect of the constraint $N_Z \leq N_0$

The respect of the constraint $N_Z \leq N_0$ implies that the values of N, N_0, π_m and π_z be such as $\Pr(N_Z > N_0) \simeq 0$. When N_Z follows a two-point distribution $\mathrm{P_T}(N_Z|N, \pi_z\pi_m)$, then N_Z takes the values $\lfloor \mathrm{A}\rfloor$ and $\lfloor \mathrm{A}\rfloor + 1$ with respective probabilities $(1-\Omega)$ and Ω, and therefore we get:

$$\Pr(N_Z > N_0) = \begin{cases} 1 & \text{if } \lfloor \mathrm{A}\rfloor > N_0 \\ \Omega & \text{if } \lfloor \mathrm{A}\rfloor = N_0 \\ 0 & \text{if } \lfloor \mathrm{A}\rfloor < N_0 \end{cases} \tag{24}$$

Hence, the respect of the constraint is ensured provided that $\lfloor \mathrm{A}\rfloor < N_0$.

References

1. Johnson NL, Kemp AW, Kotz S. Univariate discrete distributions. Third edition. Hoboken, New Jersey, USA: John Wiley & Sons; 2005.
